# LPS-induced lipid alterations in microglia revealed by MALDI mass spectrometry-based cell fingerprinting in neuroinflammation studies

**DOI:** 10.1038/s41598-022-06894-1

**Published:** 2022-02-21

**Authors:** Martina Blank, Thomas Enzlein, Carsten Hopf

**Affiliations:** 1 Center for Mass Spectrometry and Optical Spectroscopy (CeMOS), Mannheim University of Applied Sciences, Paul-Wittsack Str. 10, 68163 Mannheim, Germany; 2 Structural Molecular Biology Laboratory (LABIME), Department of Biochemistry, Federal University of Santa Catarina, Florianópolis, SC 88040-900 Brazil; 3 Medical Faculty Heidelberg, University of Heidelberg, Heidelberg, Germany

**Keywords:** Lipids, Mass spectrometry, Neuroimmunology, Microglia

## Abstract

Pathological microglia activation can promote neuroinflammation in many neurodegenerative diseases, and it has therefore emerged as a potential therapeutic target. Increasing evidence suggests alterations in lipid metabolism as modulators and indicators in microglia activation and its effector functions. Yet, how lipid dynamics in activated microglia is affected by inflammatory stimuli demands additional investigation to allow development of more effective therapies. Here, we report an extensive matrix-assisted laser desorption/ionization (MALDI) mass spectrometry (MS) whole cell fingerprinting workflow to investigate inflammation-associated lipid patterns in SIM-A9 microglial cells. By combining a platform of three synergistic MALDI MS technologies we could detect substantial differences in lipid profiles of lipopolysaccharide (LPS)- stimulated and unstimulated microglia-like cells leading to the identification of 21 potential inflammation-associated lipid markers. LPS-induced lipids in SIM-A9 microglial cells include phosphatidylcholines, lysophosphatidylcholines (LysoPC), sphingolipids, diacylglycerols and triacylglycerols. Moreover, MALDI MS-based cell lipid fingerprinting of LPS-stimulated SIM-A9 microglial cells pre-treated with the non-selective histone deacetylase inhibitor suberoylanilide hydroxamic acid revealed specific modulation of LPS-induced-glycerolipids and LysoPC(18:0) with a significant reduction of microglial inflammation response. Our study introduces MALDI MS as a complementary technology for fast and label-free investigation of stimulus-dependent changes in lipid patterns and their modulation by pharmaceutical agents.

## Introduction

Microglia play an active role in the central nervous system’s (CNS) inflammatory response. As the brain’s resident immune cells, microglia provide the first-line defense and maintain brain homeostasis supporting normal CNS development and function^1^. On the other hand, impairments in microglia activation can lead to chronic and cytotoxic inflammatory responses contributing to neuroinflammation, a pathophysiological hallmark of many neurodegenerative diseases like Alzheimer's disease (AD) and Parkinson's disease (PD)^2,3^. Therefore, current mechanism-based and potentially therapeutic approaches targeting these diseases attempt to suppress detrimental microglial inflammatory responses in an effort to delay disease progression^2,4^.

Pro-inflammatory bacterial lipopolysaccharide (LPS) stimulation, a widely used model of inflammation, drives microglial activation through the Toll-Like Receptor (TLR)-4 and nuclear factor kB (NF-κB) pathways triggering transcriptional activation of various microglial phagocytic markers, growth factors, cytokines (e.g. the interleukins IL-1β and IL-6, tumor necrosis factor (TNF)-α), nitric oxide (NO), free radicals as well as enzymes producing lipid mediators^5^. Activated microglia transcriptional signatures also comprise many lipid- and lipoprotein metabolism-associated genes, like ApoE and TREM2^6,7^ , therefore, alterations in microglia lipid metabolism as another key aspect of neuroinflammation has received much attention recently^8–10^. Indeed, it has been shown that saturated fatty acids (FA), polyunsaturated FA (PUFA) and lysophospholipids are all direct modulators of microglial activity^11–15^. Intracellular FA accumulation within lipid droplets^16,17^ and aberrant phospholipase activity (e.g. phospholipase A_2_; PLA_2_) with concomitantly increased synthesis of lipid mediators associated with microglia inflammatory response^18,19^ has also been reported. These data indicate a role of lipid metabolism in microglia activation and response, yet, our understanding of how activated microglia orchestrates lipid metabolism to mount a protective or detrimental response remains to be clarified.

Advances in the lipidomics field have allowed the investigation of physiological and pathological functions of lipids in vitro and in vivo^20^. As an emerging technology for the study of lipid metabolism in neurodegenerative states^8^, lipidomics approaches offer the possibility to track pathophysiological relevant molecules in complex cell and tissue extracts. However, lipidomics studies usually rely on the combination of liquid chromatography to mass spectrometry (MS) which requires additional extraction steps, labeling and long analysis time. Recently, we introduced fast, automatable, robust and label-free matrix-assisted laser desorption/ionization (MALDI) MS cell fingerprinting workflows^21,22^ for cellular assays capable of characterizing drug on- and off-target responses with high-throughput screening capability and minimal sample preparation.

In this study, we extended this approach to microglia research and combined it with MALDI Magnetic Resonance MS (MRMS) and Trapped Ion-Mobility (TIMS) MS to simultaneously map LPS-induced lipid pattern alterations in microglia-like cells and to identify potential lipid markers of microglial activation. Our data indicate that increased levels of sphingomyelin [SM 34:1 + K]^+^, the ceramides (Cer) [Cer d42:1 + H-H_2_O]^+^ and [HexCer d34:1 + Na]^+^, the protonated isoforms and alkali adducts of C14-, C16- and C18 lysophosphatidylcholines (LysoPC) as well as the protonated isoforms and alkali adducts of C30-, C32- and C34 phosphatidylcholines (PC), three diacylglycerols (DG) and two triacylglycerols (TG) are present in LPS-activated SIM-A9 microglia cell line. In addition, to test applicability of our workflow, when cells were pre-treated with suberoylanilide hydroxamic acid (SAHA), a known non-selective histone deacetylase (HDAC) inhibitor with reported anti-inflammatory activity^23,24^, we observed inhibition of LPS-induced IL-6 and TNF-α release as well as a specific modulation of glycerolipids (DG and TG) and LysoPC(18:0). All together, we present a proof-of-concept study for the application of whole cell MALDI MS lipid fingerprinting to the discovery of inflammation-associated lipid biomarkers in microglia and to evaluate their modulation by anti-inflammatory agents. Further understanding of the role of the microglia inflammation-associated lipid markers may provide insight into the interplay between microglia activation and lipid metabolism that could support new therapeutic options in neuroinflammatory diseases.

## Results

### MALDI-TOF MS-based cell fingerprinting detects changes in LPS-stimulated SIM-A9 microglia lipid patterns

Whole cell MALDI MS has been successfully applied to monitor biomolecular changes in mammalian cells allowing the profiling of overall content of lipids/metabolites in a label-free manner providing a snapshot of the physiological and pathological status of a biological system^21,25–29^. However, it has not yet been used for microglia studies. Therefore, in this study, we optimized our previously stablished MALDI-TOF MS-based cell fingerprinting workflow^21,30^ combining it to a platform of three synergistic MALDI MS technologies to interrogate lipid patterns in activated microglia and extract potential metabolic markers of inflammation.

**Figure 1:**
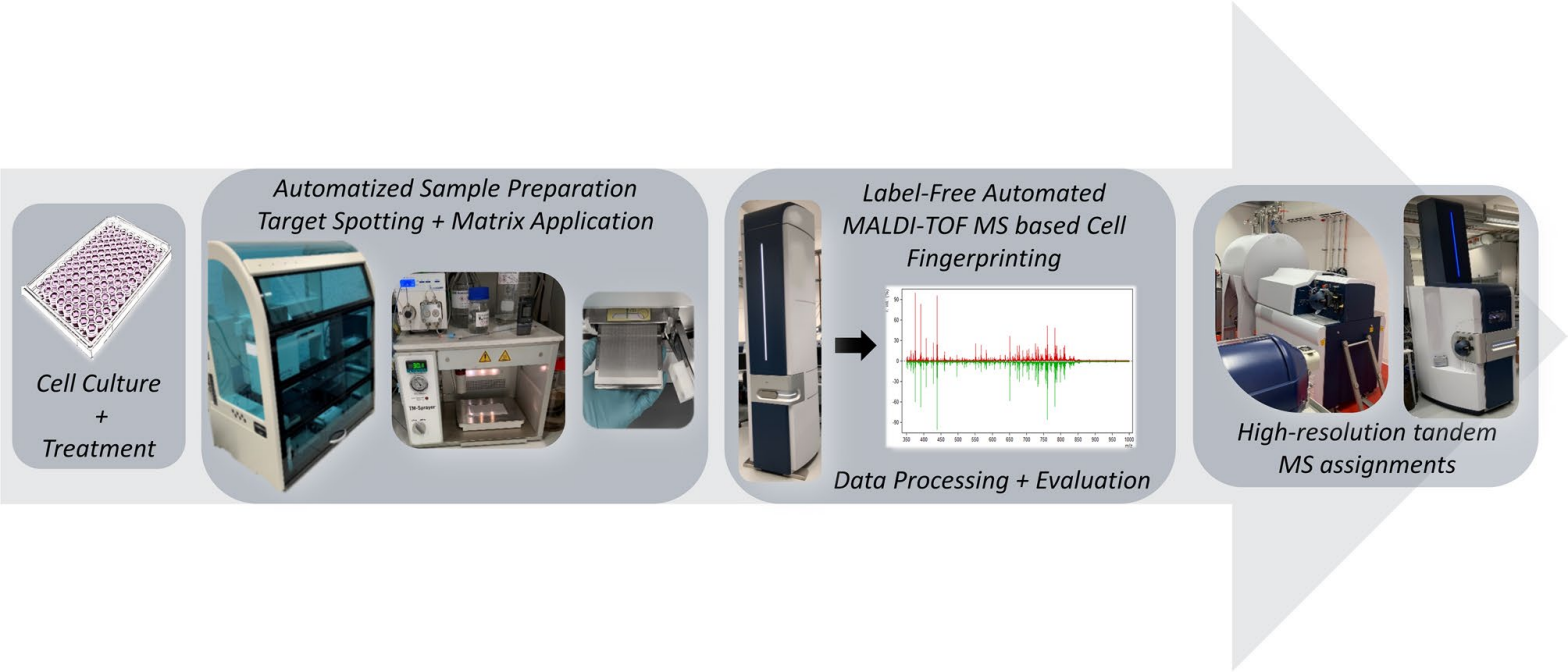
Workflow of MALDI MS microglia fingerprinting for investigation of lipopolysaccharide- (LPS-) driven changes in microglia lipids. Cultured cells are treated in 96-well format with LPS in combination with a compound or a solvent control. After removal of culture supernatants, 96-well plates are snap-frozen and stored at − 80 °C. Cells are resuspended in acetonitrile/water to 2000 cells/µl and 2 µl (corresponding to a total of 4,000 cells per spot) are spotted on a 384-well MALDI target plate in quadruplicate using a CyBio FeliX pipetting platform. DHB matrix is sprayed onto the target plate using an M3 sprayer. MS-spectra are acquired using a rapifleX MALDI-TOF mass spectrometer in automated fashion. Spectra are processed and computationally analyzed to reveal biomolecular patterns and putative markers. Ultra-high resolution mass spectrometry using a 7 T solarix XR MRMS, timsTOFfleX-MS and tandem MS are later used for identification of candidate marker lipids.

The workflow is simple, robust and reproducible starting with treatment and freezing of microglia cells that are then automatically spotted on MALDI target plates and measured with a MALDI-TOF mass spectrometer (Fig. 1). For the extraction of highly significant differential mass-to-charge ratio (*m/z*) features, processing of mass spectra includes a step of multiple-testing correction of final peak lists^31^. Significantly altered *m/z* features are then investigated by high-resolution MALDI Magnetic Resonance MS (MRMS), Trapped Ion-Mobility (TIMS) MS and tandem MS (MS/MS) for identification. The use of automation for sample preparation, MALDI MS measurements and data analysis in this workflow offered us highly defined experimental parameters to overcome both biological and experimental variances. The average Pearson's correlation coefficient of *r* = 0.92 ± 0.05 and *r* = 0.89 ± 0.06 for the replicates in this study (vehicle (VEH)-treated cells) in positive and negative ion modes, respectively, indicate a high degree of repeatability of the MS fingerprints (Suppl. Table ST1 and ST2). Finally, accurate mass and fragmentation MS/MS data used in combination with collisional cross sections (CCS) values offered increased confidence in target molecules identification.

**Figure 2:**
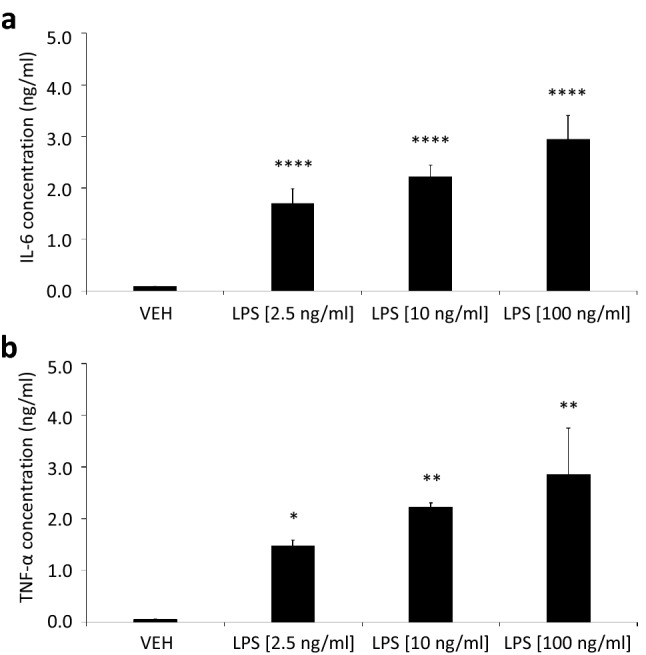
Significant SIM-A9 microglial cells cytokine secretion after LPS stimulation. Culture medium from SIM-A9 microglial cells stimulated with varying concentrations of LPS (2.5 ng/ml; 10 ng/ml; 100 ng/ml) were collected after 18 h-treatment (*N* = 2–4 per condition), and IL-6 (**a**) and TNF-α (**b**) concentrations were determined by ELISA. Data represent the mean ± s.d. One-way ANOVA **** *P* < 0.0001, ** *P* < 0.01, * *P* < 0.05 to Vehicle (VEH; 0 ng/ml).

To investigate lipid patterns associated with microglia activation, we used mouse SIM-A9 microglial cells stimulated with LPS as a model system^32^. LPS is a chemical stimulant that strongly activates microglial cells triggering rapid inflammatory responses through the TLR4 pathway promoting the release of several pro-inflammatory cytokines such as IL-6 and TNF-α^5^. Initially, to control the capacity of SIM-A9 microglial cells to mimic an effective pro-inflammatory LPS-response, we evaluated IL-6 and TNF-α release in the supernatants of LPS-stimulated cells by ELISA. Following the treatment with 2.5, 10 or 100 ng/ml of LPS for 18 h, we observed a strong (*P* < 0.0001) concentration-dependent increase in IL-6 and TNF-α release compared to VEH-control treatment corresponding to an effective activation of SIM-A9 microglial cells (Fig. 2a and b).

**Figure 3:**
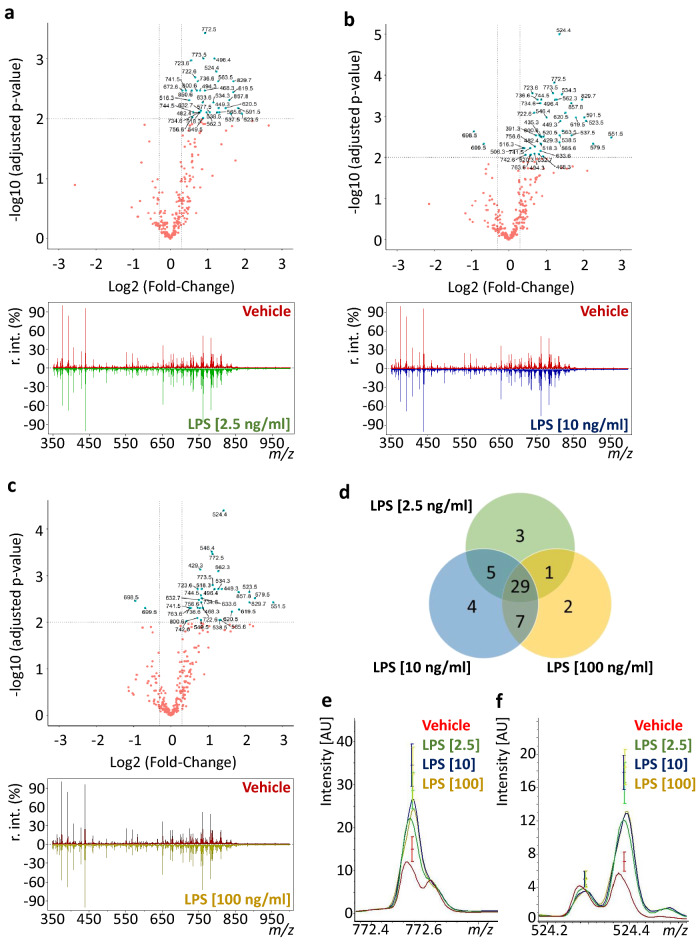
Fingerprinting of LPS-induced lipid responses in activated SIM-A9 microglial cells. (**a**)–(**c**) Log2-Fold-changes of putative lipids in VEH-treated versus LPS-stimulated microglial cells assessed by MALDI-TOF MS. Positive ion mode average mass spectra (*m/z*-range 350–1000 Da; SNR > 3) and respective volcano plots. From a total of 298 signals, the volcano plots display 38, 45 and 39 putative lipid species as significantly changed compared to vehicle control for LPS concentrations of 2.5, 10 and 100 ng/ml, respectively. Differential signals (blue) and non-differential signals (red) were determined under the conditions of log2 fold-changes ≥ 0.3 or ≤ 0.3 and Welch's T-Test and Benjamini & Hochberg adjusted p-value threshold ≤ 0.01 (*N* = 6 biological replicates with 4–8 technical replicates). (**d**) Venn diagram showing the number of *m/z* features significantly altered with up to three concentrations of LPS. (**e, f**) Averaged MALDI-TOF MS lipid fingerprints at *m/z* 772.55 (**e**) and *m/z* 524.38 (**f**) showing LPS-induced greatest alterations.

We next performed MALDI-TOF MS-based cell fingerprinting to track LPS-induced changes in SIM-A9 microglial cells lipid patterns. A total of 298 and 93 m*/z* features in positive and negative ion mode, respectively, were detected and volcano plots highlighted *m/z* features with significantly different ion intensities (TIC normalized; signal-to-noise ratio (SNR) > 3) between the VEH-treated and each LPS-treated group (Fig. 3a–c; Suppl. Fig. S1). LPS-responsive *m/z* features with adjusted *P* values ≤ 0.01 were only detected in positive ion mode. In total, we found 51 significantly altered *m/z* features. 38, 45 and 39 such *m/z* features were extracted for LPS concentrations of 2.5, 10 and 100 ng/ml, respectively (Suppl. Table ST3). Of these features, 29 (56.8%) were shared between all LPS-treated groups with increased intensities compared to unstimulated microglial cells (Fig. 3d). Among the features with greatest alterations, *m/z* 772.55 showed the highest change for LPS 2.5 ng/ml (*P*adj < 0.001; Fig. 3e and Table 1) and *m/z* 524.38 for LPS concentrations 10 and 100 ng/ml (*P*adj < 0.0001; Fig. 3f and Suppl. Table ST3). Since cells were serum starved for 6 h prior to LPS stimulation and remained without serum for the next 18 h as well as due to the use of a vehicle control these differences are not likely an effect of serum starvation or due to serum contents (e.g. exogenous metabolites). Instead, they could be rendered as prominent candidate inflammation-associated markers. Viability of SIM-A9 cells upon LPS treatment was reduced to 74% and 67% following LPS treatment with 10 and LPS 100 ng/ml, respectively. Therefore, we considered SIM-A9 microglial cells treated with LPS 2.5 ng/ml (viability 85%) for subsequent analysis and experiments (Suppl. Fig. S2). Overall, our data indicate that LPS stimulation induce significant changes in SIM-A9 microglial cell lipid patterns that can be rapid detected, and in a label-free manner, by MALDI-TOF MS cell lipid fingerprinting allowing the extraction of putative markers of inflammation.

**Table 1:** Differential lipids in LPS-treated SIM-A9 microglial cells identified using MALDI MS-based cell fingerprinting.

Observed *m/z* by TOF	LPS [2.5 ng/ml]		MS experiment level for ID	Theoretical *m/z*	Observed *m/z* by FTICR	Mass error (in ppm)	TimsTOF		Lipid assignment	Lipid Name	Class
	Adjusted *P*-value	Log2 (fold change)					*1/K*_*0*_	CCS Å^2^			
468.31	0.003	0.9	MS2	468.3084	468.3085	0.2	1.081	222.9	[LPC 14:0]^+^	LysoPC(14:0/0:0)	Lysophosphatidylcholines
482.36	0.008	0.8	MS1	482.3605	482.3605	0.0	1.123	231.3	[LPC O-16:0]^+^	LysoPC(O-16:0/0:0)	Lysophosphatidylcholines
496.35	0.001	1.2	MS2	496.3398	496.3398	0.0	1.118	230.1	[LPC 16:0]^+^	LysoPC(16:0/0:0)	Lysophosphatidylcholines
518.33	0.008	0.8	MS2	518.3217	518.3216	− 0.2	1.134	233.1	[LPC 16:0 + Na]^+^	LysoPC(16:0/0:0)	Lysophosphatidylcholines
523.48	0.008	1.9	MS1	523.4721	523.4720	− 0.2	1.216	249.9	[DG 30:0-H_2_O]^+^	–	Diacylglycerols
524.38	0.002	1.2	MS2	524.3711	524.3711	0.0	1.15	236.4	[LPC 18:0]^+^	LysoPC(18:0/0:0)	Lysophosphatidylcholines
534.31	0.005	1.2	MS2	534.2956	534.2954	− 0.4	1.137	233.6	[LPC 16:0 + K]^+^	LysoPC(16:0/0:0)	Lysophosphatidylcholines
546.37	0.010	0.9	MS2*	546.3530	546.3527	− 0.5	1.165	239.2	[LPC 18:0 + Na]^+^	LysoPC(18:0/0:0)	Lysophosphatidylcholines
562.34	0.008	1.0	MS2	562.3278	562.3278	0.0	1.170	240.1	[LPC 18:0 + K]^+^	LysoPC(18:0/0:0)	Lysophosphatidylcholines
579.55	0.010	2.2	MS2*	579.5347	579.5345	− 0.3	1.283	263.1	[DG 34:0-H_2_O]^+^	DG(16:0/18:0)	Diacylglycerols
619.55	0.004	1.7	MS2*	619.5272	619.5267	− 0.8	1.278	261.6	[DG 34:0 + Na]^+^	DG(16:0/18:0)	Diacylglycerols
632.66	0.007	0.8	MS2	632.6340	632.6337	− 0.5	1.355	277.3	[Cer d42:1-H_2_O]^+^	Cer(d18:1/24:0)	Ceramides
722.57	0.002	0.7	MS1	722.5541	722.5538	− 0.4	1.368	279.2	[HexCer d34:1 + Na]^+^	–	Ceramides
734.59	0.008	0.8	MS2	734.5694	734.5696	0.3	1.386	282.8	[PC 32:0]^+^	PC(16:0/16:0)	Phosphatidylcholines
741.55	0.003	0.4	MS2	741.5307	741.5308	0.1	1.402	286.0	[SM 34:1 + K]^+^	SM(d18:1/16:0)	Sphingomyelins
744.52	0.008	0.6	MS2	744.4940	744.4941	0.1	1.378	281.1	[PC 30:0 + K]^+^	PC(14:0/16:0)	Phosphatidylcholines
756.57	0.009	0.6	MS2	756.5513	756.5516	0.4	1.412	287.9	[PC 32:0 + Na]^+^	PC(16:0/16:0)	Phosphatidylcholines
772.55	< 0.001	0.9	MS2	772.5253	772.5254	0.1	1.407	286.8	[PC 32:0 + K]^+^	PC(16:0/16:0)	Phosphatidylcholines
800.57	0.003	0.7	MS2*	800.5566	800.5571	0.6	1.438	292.9	[PC 34:0 + K]^+^	PC(16:0/18:0)	Phosphatidylcholines
829.74	0.002	1.7	MS1	829.7256	829.7255	− 0.1	1.524	310.3	[TG 48:0 + Na]^+^	–	Triacylglycerols
857.77	0.005	1.6	MS1	857.7569	857.7577	0.9	1.545	314.4	[TG 50:0 + Na]^+^	–	Triacylglycerols

Based on volcano plots and the Venn diagram in Fig. 3d, we narrowed 29 m*/z* features (= candidate signals) increased in the LPS-treated groups down to 21 identified molecules that overlapped in all LPS groups (Welch's T-Test and Benjamini and Hochberg adjusted *P* value threshold ≤ 0.01 for positive ion mode). High resolution MS (MS1) and tandem MS (MS2) were used for assignments of candidate marker lipids. Note that, here, positional distribution of the fatty acids esterified to the glycerol backbone of the phospholipid were not determined. *MS/MS confirmation in TimsTOF fleX only.

### Identification of SIM-A9 microglia inflammation lipid-associated markers

**Figure 4:**
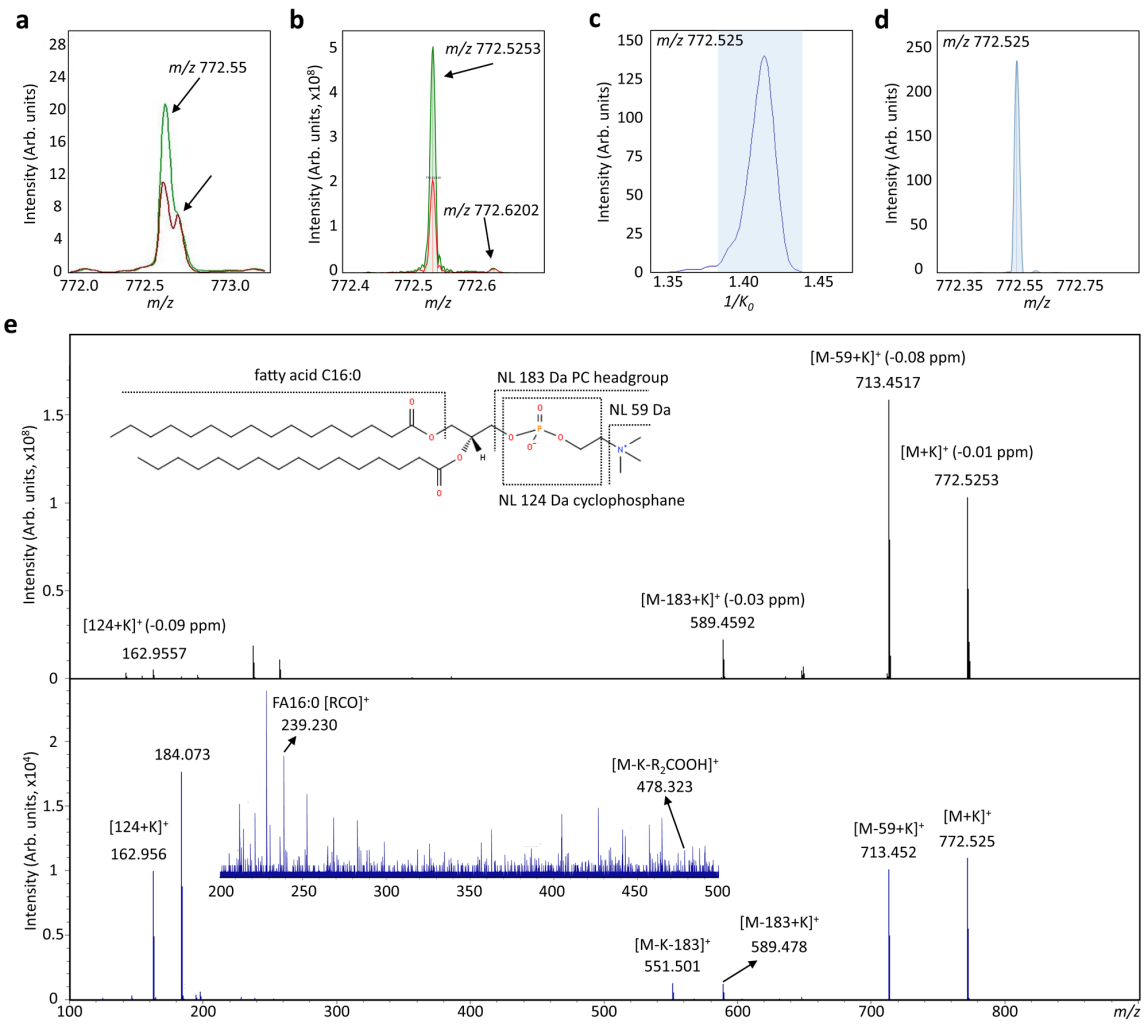
Exemplary high resolution MS spectrum and molecular structural analysis of *m/z* 772.5253 [PC(16:0/16:0) + K]^+^. (**a**) RapifleX MALDI-TOF MS-spectrum of *m/z* 772.55. (**b**) Accurate mass determination of *m/z* 772.5253 by 7 T solarix XR FTICR MS. VEH-treated (red) and LPS-treated (2.5 ng/ml; green) microglial cells. (**c** and **d**) Extracted ion mobility with 1/K_0_ = 1.407 ± 0.01 (CCS = 286.8 Å^2^) depicts the presence of only one peak for *m/z* 772.525. (**e**) Tandem MS spectra and structural elucidation of *m/z* 772.5253 by FTICR-MS (top, black) and timsTOF fleX-MS (down, blue). Typical fragments considered for the phosphatidylcholine [M + K]^+^ were the neutral loss of (CH_3_)_3_ N) [M + K-59] and [M + K − 183], the PC headgroup at *m/z* 184.0733 and cyclophosphane (124) + K^+^ at *m/z* 162.9557. Additional fragment ions at *m/z* 313 and 551 correspond to the presence of fatty acid C16:0.

Next, to characterize the prominent candidate markers molecules, we first remeasured the *m/z* signals using an Ultra-high resolution SolariX FTICR MS. In total, the identity of 21 out of the 29 potential inflammation-associated markers could be assigned based on stringent accurate mass determination (∆*m/z* < 1 ppm) (Table 1). The remaining unassigned *m/z* features were either isotopic peaks or unavailable in the database (Suppl. Table ST3). 12 out of the 21 m*/z* species were later confirmed by high resolution MS/MS experiments with fragmentation patterns (Fig. 4e and Suppl. Figs. S3–S7, S9 and S11–S16) corresponding to structure-specific characteristic ions compared to the literature^33^. MS and MS/MS measurements of lipids were also done with timsTOFfleX which enabled the confirmation of 4 more lipid species (Fig. 4e and Suppl. Figs. S3–S17) and determination of collisional cross sections (CCS) values of ions. Here, CCS values were used as an additional molecular descriptor to confirm and correctly assign lipid markers. Although available CCS values libraries are mainly based on electrospray ionization generated data and CCS values for [M + K] ^+^ are scarce, our experimental CCS values were in good agreement (average deviation < 1%) with those predicted and previously reported in CCS databases and the literature where available (Suppl. Table ST4)^34–38^.

Representative MALDI-TOF MS, high-resolution MS spectrum and timsTOFfleX spectra with the tandem MS analysis are provided for *m/z* 772.55 in Fig. 4. The mean MALDI-TOF MS spectra for VEH and LPS-treated microglial cells shows the upregulation of the ion at *m/z* 772.55 with a neighboring unresolved peak (Fig. 4a). High-resolution FTICR MS allowed to distinguish both *m/z* values and assign the mass 772.5253 as [PC 32:0 + K]^+^ with high accuracy (< 1 ppm error) (Fig. 4b). Additionally, the extracted ion mobility with 1/K_0_ = 1.407 ± 0.01 (CCS = 286.8 Å^2^) revealed the occurrence of one feature under for *m/z* 772.525 (Figs. 4c and d). Collision-induced dissociation (CID) of the precursor ion *m/z* 772.525 in both FTICR-MS and timsTOFflex-MS (Fig. 4e) confirm that the feature is the potassium adduct of PC(32:0). Characteristic fragment ions of PC lipid species are the prominent ions at *m/z* 713.45 (neutral loss of 59) and 589.47 (neutral loss of 183) as well as *m/z* 162.95 which confirm the potassium adduct (124 cyclophosphane + K)^33^. Lastly, the fragment ions at *m/z* 313 and 551 suggest the presence of one fatty acid chain as C16:0. Therefore, the main component of the detected ion at *m/z* 772.525 is attributed as potassium adduct of PC (16:0/16:0).

In summary, our multiplatform MALDI MS-based cell lipid fingerprinting analysis, enabled the confident assignment based on accurate mass and tandem MS fragmentation of three sphingolipids [SM 34:1 + K]^+^, [Cer d42:1-H_2_O]^+^ and [HexCer d34:1 + Na]^+^; eight protonated isoforms and alkali adducts of C14-, C16- and C18 lysophosphatidylcholines (LysoPC); the protonated isoform of C32- and four alkali adducts of C30-, C32- and C34 phosphatidylcholines (PC); as well as five glycerolipids [DG 30:0-H_2_O]^+^, [DG 34:0-H_2_O]^+^, [DG 34:0 + Na]^+^, [TG 48:0 + Na]^+^ and [TG 50:0 + Na]^+^ (Table 1). Together, these lipids represent a potential panel of discriminant metabolic markers in activated microglia that should be considered in further validation studies, to increase mechanistic understanding of therapeutics and to be monitored in early drug development.

### SAHA inhibit cytokines secretion and specifically modulate LPS-stimulated SIM-A9 microglia glycerolipids response and LysoPC(18:0)

**Figure 5:**
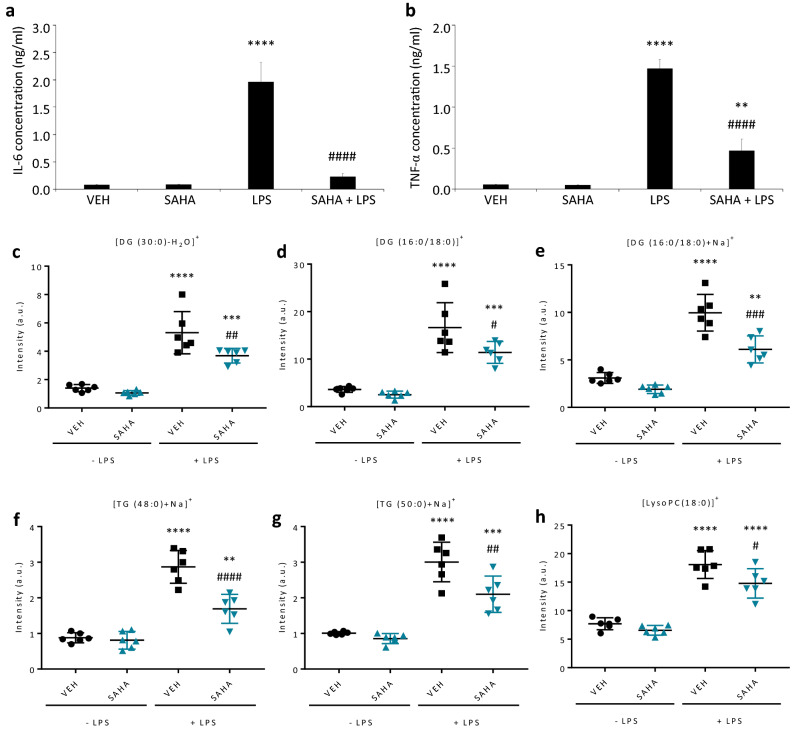
SAHA treatment reduce IL-6 and TNF-α release and modulate inflammation-associated lipid markers in LPS-stimulated SIM-A9 microglial cells. SIM-A9 microglial cells were treated for 1 h with SAHA or vehicle control (VEH) prior to LPS-stimulation for 18 h (LPS 2.5 ng/ml). Culture supernatants were collected, IL-6 (**a**) and TNF-α (**b**) release was assessed by ELISA (*N* = 2–4 per condition). Data represent the mean ± s.d. One-way ANOVA with Tukey’s post hoc test. **** *P* ≤ 0.0001 and ** *P* ≤ 0.01 to Vehicle; ### *P* ≤ 0.0001 to LPS-stimulated cells without HDAC inhibitor. (**c-h**) Effect of SAHA on LPS-induced lipid levels. MALDI-TOF mass spectra of cells were acquired using a rapifleX. One-way ANOVA with Tukey’s post hoc test. **** *P* ≤ 0.0001; *** *P* ≤ 0.001 and ** *P* ≤ 0.01 indicates a significant difference from VEH via Tukey’s post hoc test. # *P* ≤ 0.05; ## *P* ≤ 0.01; ### *P* ≤ 0.001 and #### *P* ≤ 0.0001 indicates a significant difference from LPS via Tukey’s post hoc test. Data are expressed as mean ± s.d. (*N* = 6 biological replicates with 4 technical replicates each). DG (Diacylglycerol), TG (Triacylglycerol) and LysoPC (Lysophosphatidylcholine).

Once the automated MALDI MS cell-based fingerprinting setup was established and inflammation-associated lipid markers were identified, we next sought to test the general applicability of our workflow by investigating the therapeutic potential of the broad spectrum HDAC inhibitor SAHA for the modulation of LPS-dependent lipid inflammation responses in microglial cells (Fig. 5). Emerging data have shown the beneficial effects of HDAC inhibitors in inflammation-related diseases^39–43^ and recent research supports the interplay between lipid metabolism and lysine acetylation during inflammation responses^20,44,45^ with acetyl coenzyme A (acetyl-CoA), a key intermediary metabolite in lipid metabolism, being the main substrate for lysine acetylation by histone acetyltransferases (HATs)^45,46^. On the contrary, HDAC-containing protein complexes catalyze the removal of N-acetyl groups from lysine residues^47^ which activity and expression is increased during microglial activation and particularly important for TLR-activated inflammatory responses^23,48^. Therefore, we hypothesized that anti-inflammatory action of SAHA might also involve changes in lipid metabolism.

We found that 1 h pre-treatment with SAHA (1 µM) completely abrogated the LPS-induced IL-6 release and significantly reduced LPS-induced TNF-α levels in SIM-A9 microglial cells compared to VEH-treated cells (Figs. 5a and b). Interestingly, MALDI-TOF MS cell-based lipid fingerprinting of LPS-stimulated microglial cells pretreated with SAHA revealed specific modulation of inflammation-associated glycerolipids. LPS-induced [DG(30:0)-H_2_O]^+^, [DG(16:0/18:0) + Na]^+^ and [TG(48:0) + Na]^+^ and [TG(50:0) + Na]^+^ levels were strongly attenuated by SAHA treatment (*P* = 0.010; *P* < 0.001, *P* < 0.001 and *P* = 0.002, respectively compared to LPS-treated cells; Figs. 5c, e–g). Significative attenuation of [DG(16:0/18:0) + H]^+^ (*P* = 0.024 compared to LPS-treated cells was also observed. Additionally, a noteworthy modulation of LysoPC(18:0) was detected (*P* = 0.027, Fig. 5h). Cell viability was confirmed by MTT assay (Suppl. Fig. S18). Altogether, these results demonstrated that SAHA significantly lowered the LPS-induced inflammatory cytokine response in SIM-A9 microglial cells which was accompanied by specific modulation of some inflammation-associated lipid markers indicating glycerolipids metabolism as possible off-target pathway for the SAHA anti-inflammatory effect.

## Discussion

In this work, we present a multiplatform MALDI MS-based cell lipid fingerprinting approach to explore inflammation-associated lipid markers in LPS-stimulated microglia-like cells. To the best of our knowledge, no study has been reported on the use of whole cell MALDI MS to interrogate lipid patterns in activated microglia. Together, the current findings reveal that microglia inflammation-evoked activation of TLR4 leads to significant changes in the cell’s lipid pattern with alterations in major lipid pathways such as the sphingolipid (SM and Cer species), glycerophospholipid (LysoPC and PC species) and glycerolipid (DG and TG species) metabolism pathways corroborating previous microglia lipidomics studies^12,17^.

LPS treatment is the most employed activation strategy in microglia studies with well understood signaling processes inducing transcription and expression of proinflammatory and neurotoxic genes^5,48^. Commonly used methods to monitor microglia activation are the determination of changes in levels of activation markers by ELISA, fluorescence assays, western blots and quantitative RT-PCR^49^. However, microglia activation also leads to metabolic reprogramming and changes in lipid homeostasis^10,12^. Therefore, our MALDI MS-based cell lipid fingerprinting strategy represents a further potential tool to investigate microglia activation and detect inflammation-associated metabolic alterations. A key feature of our untargeted workflow is that we initially perform an unbiased exploration of *m/z* features from LPS-stimulated cells which allow the rapid detection and extraction of a panel of highly significant differential lipid markers. The combination with high-resolution MS and tandem MS supports the identification of such markers, which can then be further validated and used to probe multiple targets of small-molecule inhibitors of microglial cell activation.

Exacerbated brain inflammatory responses are present in many neurodegenerative diseases^3^. With the current knowledge on lipids as important mediators of immune responses^20,50^, differentially expressed lipids in activated microglia can be potentially used as markers for neuroinflammation. It has been demonstrated the involvement of LysoPCs, PCs, Ceramides and Sphingomyelins in microglial inflammatory responses^51–53^. Particularly in AD, characteristic lipids, such as LysoPC(16:0), LysoPC(18:0), LysoPC(18:1) and Ceramides, were found associated with beta-amyloid plaques^54^. In like manner, these LysoPCs were also found up-regulated in a PD mouse model brain^55^. Accordingly, we found increased levels of LysoPC(14:0), LysoPC(16:0) and LysoPC(18:0) as well as Ceramides in activated microglia-like cells. As a response to an inflammatory stimulus, pro-inflammatory cytokines can activate PLA_2_ and Phosphatidylcholine-specific phospholipase C -mediated processes as well as Sphingomyelinases to hydrolyze membrane PCs and SMs generating LysoPCs and Cer, respectively^20,56^. Increased PLA_2_ activity in microglial cells upon LPS-treatment, which may be related to higher levels of LysoPCs, have also been demonstrated^18,57^. Remarkably, LysoPCs and Ceramides are well known cell lipid mediators that act as second messengers having both pro‐survival and death roles triggering alterations in mitochondrial function, oxidative stress and gene transcription with a central role in inflammation signaling^11,14,51,53,58,59^.

Our whole-cell MALDI MS approach was also capable of detecting LPS-induced alterations in glycerolipids (DG and TG species). Accordingly, accumulation of DG was demonstrated in AD and PD brains^60–62^, in activated murine macrophages^63^ and in LPS-stimulated BV2 cells^12,17^. DGs are minor components of the lipid bilayer that can also act as glycerophospholipid-derived lipid mediators in response to immune activation^64^. As a second messenger, it regulates activation of many protein kinases (PK), such as PKC and DG kinases (DGK), directly modulating nuclear signal transduction^64,65^. Ceramide signal may also be converted to one that is mediated by DG through the action of sphingomyelin synthases that breaks PC species releasing DG while adding a PC headgroup to ceramide to form SM^56^. Besides, DG in the lipid biosynthesis serve as precursors for the major glycerophospholipids, like PC, as well as for TG synthesis^66^. We also found increased levels of two TGs [TG(48:0) and TG(50:0)] in LPS-activated murine SIM-A9 microglial cells. This finding is consistent with recent reports that demonstrated higher levels of TG(48:0) and TG(50:0) and accumulation of TG-enriched lipid droplets in LPS-treated microglial cells^12,17^. TG is the main component of lipid droplets involved in energy storage and in the sequestration of FAs to reduce their availability for the activation of several signaling pathways (e.g. TLR and NF-κB inflammatory pathways, activation of protein phosphatase 2A (PP2A) by ceramides or of PKC by DG) protecting cells from injury as well as in storing lipid precursors to regulate signaling pathways^50,67^. Moreover, the metabolic reprogramming of activated microglia involves changes in mitochondrial function and in the tricarboxylic acid (TCA) cycle that can result in increased production of TG within lipid droplets^16,17,68^. In this way, our observations further corroborate an involvement of glycerolipids in TLR4-dependent inflammation response in microglia-like cells and also support the use of our MALDI MS-based cell lipid fingerprinting approach to detect alterations in activated microglia lipid metabolism.

Here, we also showed that modulation of acetylation by blocking HDACs activity with SAHA profoundly impaired LPS-induced pro-inflammatory cytokines in SIM-A9 microglial cells and partially rescued LPS-induced glycerolipid levels, as detected by our MALDI-TOF MS-based cell lipid fingerprinting. Lysine acetylation regulates the activity of intermediate metabolism enzymes of the FA pathways and of the TCA cycle^69^ with emerging evidences on inflammation-associated metabolic diseases and aging supporting a multilayered lipid-epigenetic interplay^70–72^. Our findings are consistent with reports showing a decrease of LPS-induced cytokine expression after SAHA treatment in microglial cells, mixed glial cultures and macrophages^23,42,43,73^ as well as with studies of lipid accumulation inhibition by SAHA in hepatic cells^74^ and in cultured adipocytes^75^ and by MS-275 (class I-specific HDAC inhibitor) in human macrophages^39^. A reduction in DG levels can sign a shift to a beneficial metabolic state since its generation is associated to inflammatory response as previously reported^61,64^. Additionally, the reduction in TGs may indicate an attempt of the cells to restore energy metabolism and cell membrane synthesis along with regulation of inflammatory signaling pathways through release of FAs by TG lipolysis from LPS-induced lipid droplets^9,67,68^, an aspect that requires additional investigation. Another noteworthy finding from the current study was the specific effect of SAHA on LPC(18:0) indicating involvement of this particular LysoPC with the anti-inflammatory effects observed which may indicate regulation of PLA_2_ activity^18,57^ and/or of LPC acyltransferase activity, an enzyme that converts LysoPCs back to PC in the presence of acetyl-CoA^76^. Nevertheless, follow-up studies are needed to verify these observations and validate these potential markers.

Overall, we present here a comprehensive MALDI MS-based cell lipid fingerprinting workflow for microglia research by showing the modulation of inflammatory lipid-associated markers in LPS-stimulated microglia-like cells. Our workflow also displays promise application for the investigation of lysine acetylation involvement in neuroinflammation through the use of HDAC tool inhibitors and bring to focus the cross-talk between lipid pathways, inflammation and lysine acetylation in microglial cells. Furthermore, our combined label-free automated MALDI MS-based cell fingerprinting approach is robust and reproducible with little sample preparation which can be scaled-up to enable high-throughput and support drug screening in neuroinflammation as well as contribute to the understanding of the role of lipids in microglia.

## Materials and methods

### Chemicals

All reagents were of HPLC or cell culture grade. Lipopolysaccharide from *Escherichia coli* O55:B5 (LPS, Cat. No. L2880) was purchased from Sigma-Aldrich (Taufkirchen, Germany). Phosphate buffer saline (PBS; Biowest, Cat. No. L0615), Acetonitrile (ACN; Merck, Cat. No. 100030), Trifluoracetic acid (TFA; Merck, Cat.Nno. 108262), 3-(4,5-Dimethyl-2-thiazolyl)-2,5-diphenyltetrazoliumbromid (MTT; PanReac AppliChem, Cat. No. A2231) and Dimethylsulfoxide (DMSO; Cat. No. 23500260) were purchased from VWR International (Darmstadt, Germany). Milli-Q water (ddH_2_O; Millipore) was prepared in-house. Mouse IL-6 and TNF-α ELISA kits (Cat. No. 900-TM50 and 900-TM54) were obtained from PeproTech GmbH (Hamburg, Germany). 2,5-dihydroxybenzoic acid (DHB; Cat. No. 8201346) was purchased from Bruker Daltonics (Bremen, Germany). Brain Total Lipid Extract (TBLE; Cat. No. 131101) was from Avanti Polar Lipids (Alabaster, USA). SAHA (suberoylanilide hydroxamic acid, Vorinostat; Cat. No. 10009929) was purchased from Cayman Chemical (Ann Harbor, USA). ESI-L LC/MS tuning mix (Cat. No. G1969-85,000) was from Agilent (Santa Clara, USA).

### Cell culture and treatment in 96-well format

SIM-A9 mouse microglial cells (ATCC Cat# CRL-3265, RRID:CVCL_5I31) were cultured in DMEM/F12 (ATCC^®^ 30–2006™) supplemented with 1% Penicillin/streptomycin (PEN/STREP; Cat. No. 15140122), 10% heat-inactivated fetal bovine serum (FBS, Gibco, Cat. No. 10500064), and 5% heat-inactivated horse serum (FHS, Gibco, Cat. No. 26050088)^77^. Cells were incubated at 37 °C in humidified air containing 5% CO_2_.

SIM-A9 cells were seeded 0.2 × 10^6^ per mL in 96-well plates and incubated for 24 h. Medium was changed to serum-free medium for 6 h before treatments. For LPS treatment without SAHA, cells were treated with PBS (vehicle; VEH) or stimulated with LPS diluted in PBS (2.5, 10 or 100 ng/ml) for 18 h. For LPS treatment in the presence of SAHA, cells were treated for 1 h with compound or DMSO prior to LPS-stimulation for additional 18 h (LPS 2.5 ng/ml). SAHA was dissolved in DMSO as stock solution and the final concentration of DMSO in the medium was kept under 0.1%. The inhibitor concentration was chosen based on known IC_50_ and previous reports^24^. Culture supernatants were stored at − 80 °C for ELISAs. Cells were washed once with cold PBS and the 96-well plate was snap-frozen in liquid nitrogen and stored at -80 °C until use. Cytokine levels in culture medium collected from LPS-stimulated cells and/or cells treated with inhibitor with or without LPS were measured by ELISA according to the manufacturer's instructions. Absorbance was read at 450 nm on a spectrophotometer (Multiscan Spectrum, Thermo Fisher Scientific, Germany), and IL-6/TNF-α concentrations calculated based on calibration curves.

### Automated MALDI-TOF mass spectrometry

Automated preparation of cells on MALDI MS target plates (MTP) was done with a CyBio^®^ FeliX pipetting platform (Analytik Jena AG, Germany). 96-well plates with frozen cells were taken out of the − 80 °C freezer, and cells were immediately resuspended at ~ 2,000 cells per µl in ddH_2_O/ACN (70:30). 2 µl were applied onto ground steel MTP (Bruker Daltonics, Bremen, Germany). Four technical replicates of each plate well were prepared during transfer to a 384-well MTP. After air drying of sample spots, 20 mg/ml DHB-matrix in ACN/ddH_2_O (50:50) supplemented with 2.5% TFA was sprayed using an HTX M3 Sprayer (HTX Technologies Carrboro, USA). The spray protocol included a 50 °C spray nozzle temperature with 60 µl/min matrix flow rate. Four layers of matrix were sprayed with a spray-head velocity of 1000 mm/min and the distance between sprayed lines was 2 mm^21,22^.

MTPs were measured on a rapifleX MALDI-TOF MS (Bruker Daltonics, Bremen, Germany). The FlexControl 4.0 AutoXecute tool (Bruker Daltonics) was used to perform automated data acquisition. Measurements were done in reflector-positive and -negative ion modes in a mass range of *m/z* 300 − 1800. The acquisition mode was set to random walk, and 6000 laser shots were accumulated in 60 shot steps per spot using 10 kHz laser frequency. External calibration was performed using the TBLE calibration mixture spotted in close proximity to the sample spots. All spots from the same assay were measured on the same day. Samples originating from different passage numbers of cultured cells that were analyzed on different days are referred to as biological replicates.

### MALDI-FTICR and MALDI-timsTOF mass spectrometry

MALDI-Magnetic Resonance MS (MRMS; solariX 7 T XR; Bruker Daltonics, Bremen, Germany) and MALDI-Trapped Ion-Mobility Time-of-Flight (timsTOF) MS (timsTOFfleX; Bruker Daltonics, Bremen, Germany) were used for verification of peak identity of marker molecules.

Ultra-high resolution (R ~ 500,000 at *m/z* 400) MR mass spectra were acquired with ftmsControl 2.1.0 (Bruker Daltonics, Bremen, Germany) in positive-ion mode within the *m/z* range 150–3000 using a 4 M AMP transient mode. Calibration was performed externally using the TBLE calibration mixture and a single-point on-line internal calibration was performed on the PC peak at *m/z* 760.5851 [PC(34:1) + H]^+^ with a mass tolerance of 5 ppm. MALDI parameters were optimized to maximize intensity and resolution^21^. After full scan recordings, MS/MS spectra were acquired by CID fragmentation of candidate lipid markers.

Additionally, full scan MS and MS/MS spectra were recorded on the timsTOFfleX mass spectrometer to assist with molecular identification^78,79^. Spectra were acquired in both qTOF and TIMS-on modes of operation in positive ion mode within the *m/z* range 100 – 1000. The qTOF was calibrated using “quadratic enhanced”-fit and the TIMS dimension was calibrated linearly using selected ions from the ESI LC/MS tuning mix (*m/z*, CCS: 118.0862, 120.8; 322.0481, 152.8; 622.0289, 201.6 and 922.0097, 241.8) in positive mode. MALDI parameters were optimized to maximize intensity and resolution. The ion mobility was scanned from 0.6 to 1.70 Vs/cm^2^. Fragmentations were performed by CID with a collision energy ranging from 40 to 55 eV.

### Processing of mass spectra and data analysis

Initial manual recalibration of mass spectra was done in flexAnalysis 4.0 software (Bruker Daltonics, Bremen, Germany). Quadratic calibration was performed internally using the lysophosphatidylcholine LysoPC(16:0) ([M + H]^+^ *m/z* 496.339) and PC(34:1) ([M + H]^+^ *m/z* 760.585, [M + Na]^+^ *m/z* 782.567 and [M + K]^+^ *m/z* 798.541) for positive ion mode mass spectra. ClinPro Tools 3.0 software (Bruker Daltonics, Bremen, Germany) was used for spectra grouping, mean spectra visualization and peaks calculation with the following parameters: TIC normalization; Resolution of 10,000; TopHat baseline subtraction with 10% Minimal Baseline Width; Savitzky − Golay Spectra smoothing with 0.1 m*/z* width and 1 cycle; Null spectra and Noise spectra exclusion enabled. Peaks were picked on total average spectra with SNR thresholds of three (See supplementary methods for processing of negative ion mode mass spectra).

Final peak lists were loaded into R 4.0.2 environment (RFoundation for Statistical Computing, Vienna, Austria, http://www.R-project.org)^80^. Readxl (https://cran.r-project.org/package=readxl) and tidyverse (https://cran.r-project.org/package=tidyverse) packages were used to read and manipulate data while ggpubr (https://cran.r-project.org/package=ggpubr) together with ggrepel (https://cran.r-project.org/package=ggrepel) were used to plot the data^21^. Fold-changes of putative lipids in VEH-treated versus LPS-stimulated microglial cells were determined by Welch's T-Test with Benjamini & Hochberg adjusted *P*-value threshold ≤ 0.01.

All data represent the mean ± s.d. unless indicated otherwise. Unpaired T-test was used to compare two groups and One-way ANOVA with Tukey’s post hoc test for multiple comparisons. *P-*values ≤ 0.001, ≤ 0.01 and ≤ 0.05 were used to indicate highly statistically significant, very statistically significant and statistically significant, respectively.

For FTICR and timsTOF MS data visualization and evaluation, the Bruker Compass DataAnalysis 5.3 (Bruker Daltonics, Bremen, Germany) was used. Lipid identifications were determined based on mass accuracy using the LIPIDMAPS lipidomics gateway [lipidmaps.org;^81^] and on the fragmentation patterns observed in MS/MS spectra, when available^33^. In positive mode, characteristic ions for LysoPC and PC lipid species were: the neutral loss (NL) of trimethylamine (CH_3_)_3_ N) [M-59] and choline phosphate [M-183] with presence of the PC head group ion at *m/z* 184.073, as well as the cyclophosphane ion at *m/z* 146.981 or *m/z* 162.955, in case of sodium and potassium adducts, respectively. The characteristic ion for sphingomyelin (SM) in positive mode considered was the *m*/*z* 184.073 and other possible ions were the water loss [M + H − 18]^+^, ions at *m/z* 264 and 280 corresponding to the long chain base and fatty acid in the molecule and [M-59] and [M-183]. Ceramides (Cer) predominant fragment ion corresponds to the loss of a water molecule [M + H − 18]^+^ and characteristic ions correspond to the sphingoid bases, e.g. at *m/z* 264 and 282 for Cer species containing sphingosine^33^. TimsTOF fleX reduced ion mobility values (1/K_0_) were converted to collisional cross section (CCS) values using the Bruker Compass Mobility Calculator.

## Supplementary Information

Supplementary Information.

## Data Availability

There is currently no public data repository for MALDI-MS fingerprinting data. However, key raw data used in this paper are available at figshare: 10.6084/m9.figshare.17205908. Additional data are available from the corresponding author upon reasonable request.

## Code availability

The in-house R scripts are publicly available on the CeMOS GitHub at https://github.com/CeMOS-Mannheim/Blank2021.
