# Supplementary material for: LPS-induced lipid alterations in microglia revealed by MALDI mass spectrometry-based cell fingerprinting in neuroinflammation studies
Source: Sci Rep. 2022 Feb 21;12:2908. doi: 10.1038/s41598-022-06894-1 (PMC8861089; doi:10.1038/s41598-022-06894-1)
Supplement: Supplementary file 1 — Supplementary Information. [file 41598_2022_6894_MOESM1_ESM.pdf]

Supplementary Information

LPS-induced lipid alterations in microglia revealed by MALDI Mass Spectrometry-based cell fingerprinting in neuroinflammation studies

Martina Blank, Thomas Enzlein, Carsten Hopf

Table of contents

Supplementary Methods	Description of methods used for supplementary figures
Figure S1	Screening of differential expressed lipids between VEH and LPS-stimulated microglia cells by MALDI-TOF MS in the negative ion mode.
Figure S2	Percentage of viable cells after LPS-treatment.
Figure S3 – S17	FTICR and/or TimsTOF MS/MS spectra of inflammation-associated lipid markers in activated microglia.
Figure S18	Percentage of viable cells after treatment with SAHA.
Table ST1	Pearson's correlation for the vehicle-treated microglial cells MALDI-TOF MS fingerprinting in positive ion mode.
Table ST2	Pearson's correlation for the vehicle-treated microglial cells MALDI-TOF MS fingerprinting in negative ion mode.
Table ST3	Significantly altered m/z features in LPS-treated microglial cells.
Table ST4	Comparative CCS values of significantly altered m/z features in LPS-treated microglial cells.

Supplementary Methods

Cell Viability Assay

Cell viability was measured via MTT assay in parallel experiments. Briefly, MTT reagent (5mg/ml in PBS) was added to cells after 24h in culture, and cells were incubated for 2 hours. The reaction was stopped with 10 % sodium dodecyl sulfate (w/v) in DMSO supplemented with 50 % 0.01 M hydrochloric acid). After 2 h incubation absorbances were measured at 540 nm and 630 nm, for background correction, using a plate reader (Multiscan Spectrum, Thermo Fisher Scientific, Schwerte, Germany). Wells without cells were used as blanks.

Processing of MALDI-TOF MS mass spectra and data analysis in negative ion mode

To verify differences in vehicle (VEH)-treated versus lipopolysaccharide (LPS)-stimulated microglia cells, the workflow for MALDI-TOF MS data processing and analysis consisted of an initial manual recalibration of mass spectra in flexAnalysis 4.0 software (Bruker Daltonics). Quadratic calibration was performed internally using the phosphatidylethanolamine PE(36:1) ([M-H]- m/z 744.554), the phosphatidylinositol PI(36:4) ([M-H]- m/z 857.517), PI(38:4) ([M-H]- m/z 885.549) and the Sulfatide C24:1 ([M-H]- m/z 888.624). Further processing was done in ClinPro Tools 3.0 software (Bruker Daltonics,) where spectra grouping was performed with mean spectra visualization and peaks calculated with the following parameters: TIC normalization; Resolution of 10,000; TopHat baseline subtraction with 10% Minimal Baseline Width; Savitzky−Golay Spectra smoothing with 0.1 m/z width and 1 cycle; Null spectra and Noise spectra exclusion enabled. Peaks were picked on total average spectra with signal-to-noise (S/N) thresholds of five.

Supplementary Data

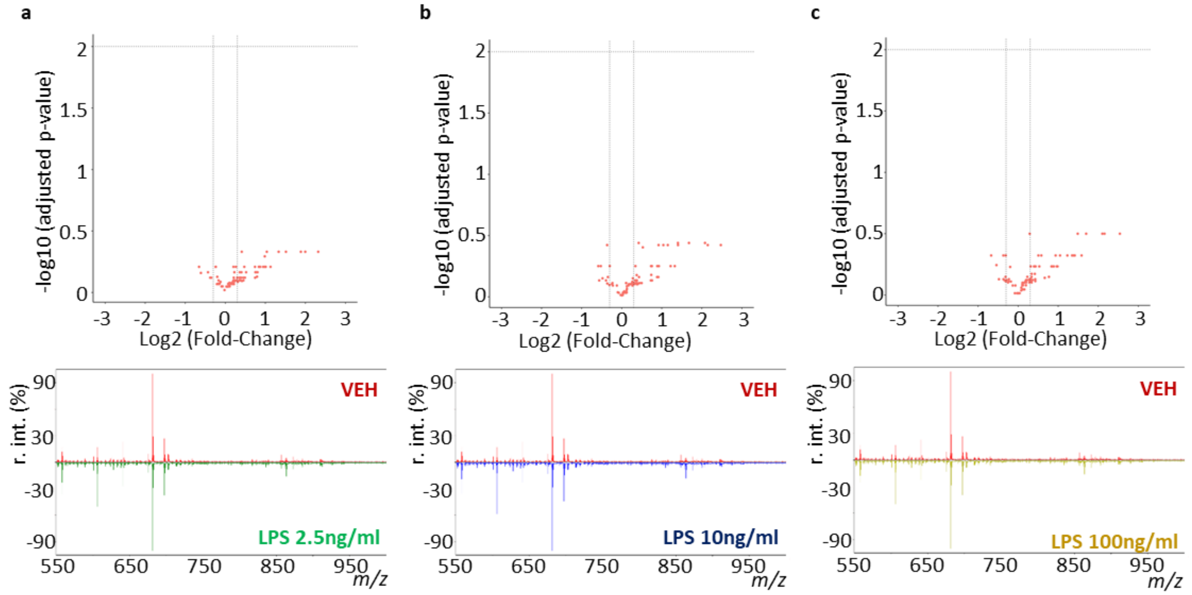

Figure S1. Screening of differential expressed lipids between VEH and LPS-stimulated microglia cells by MALDI-TOF MS in the negative ion mode. Negative ion mode average mass spectra (m/z range 550-1000 Da; S/N > 5) and respective volcano plots are presented. After the Welch's T-Test and Benjamini & Hochberg adjusted *P* value threshold of ≤ 0.01, from the total of 93 signals no differential signal between VEH-treated and LPS-treated microglial cells could be determined. The volcano plots show non-differential signals (*red*) in log2folding change of ≥0.3 or ≤ 0.3 and log10 folding *P*-values for cells treated with LPS 2.5 ng/ml (a), LPS 10 ng/ml (b) and LPS 100 ng/ml (c) compared to the vehicle group (N = 4 biological replicates).

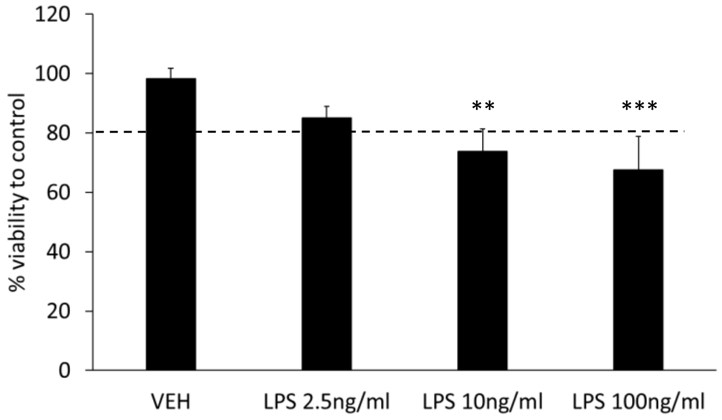

Figure S2. Percentage of viable cells after LPS-treatment. SIM-A9 microglial cells were treated with varying concentrations of LPS (2.5 ng/ml; 10 ng/ml; 100 ng/ml) for 18h, and cell viability was assessed by MTT assay. Data represent the mean of N = 4 ± s.d. One-way ANOVA with Tukey’s post hoc test. ** P <0.01 and *** P<0.001 to Vehicle (VEH).

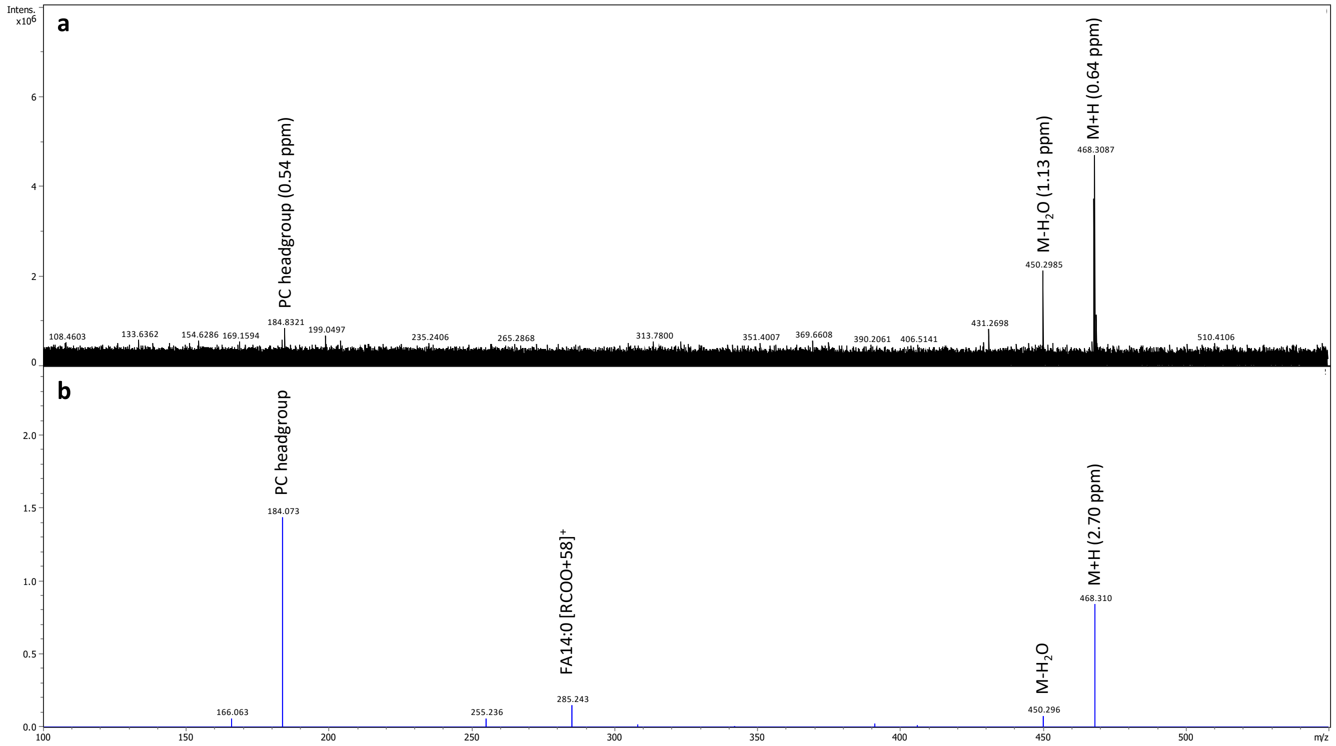

Figure S3. FTICR (a) and TimsTOF (b) MS/MS spectra of *m/z* 468.3087 [LPC 14:0]$^+$

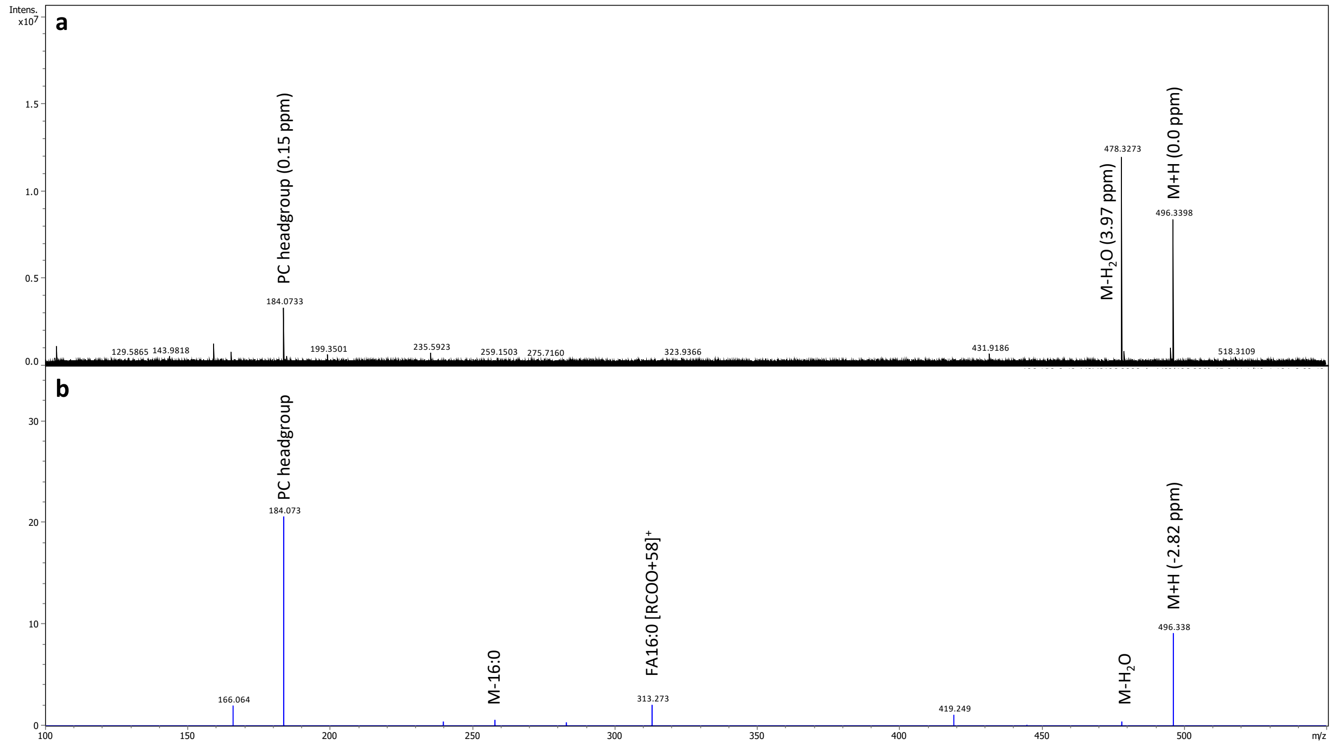

Figure S4. FTICR (a) and TimsTOF (b) MS/MS spectra of *m/z* 496.3398 [LPC 16:0]$^+$

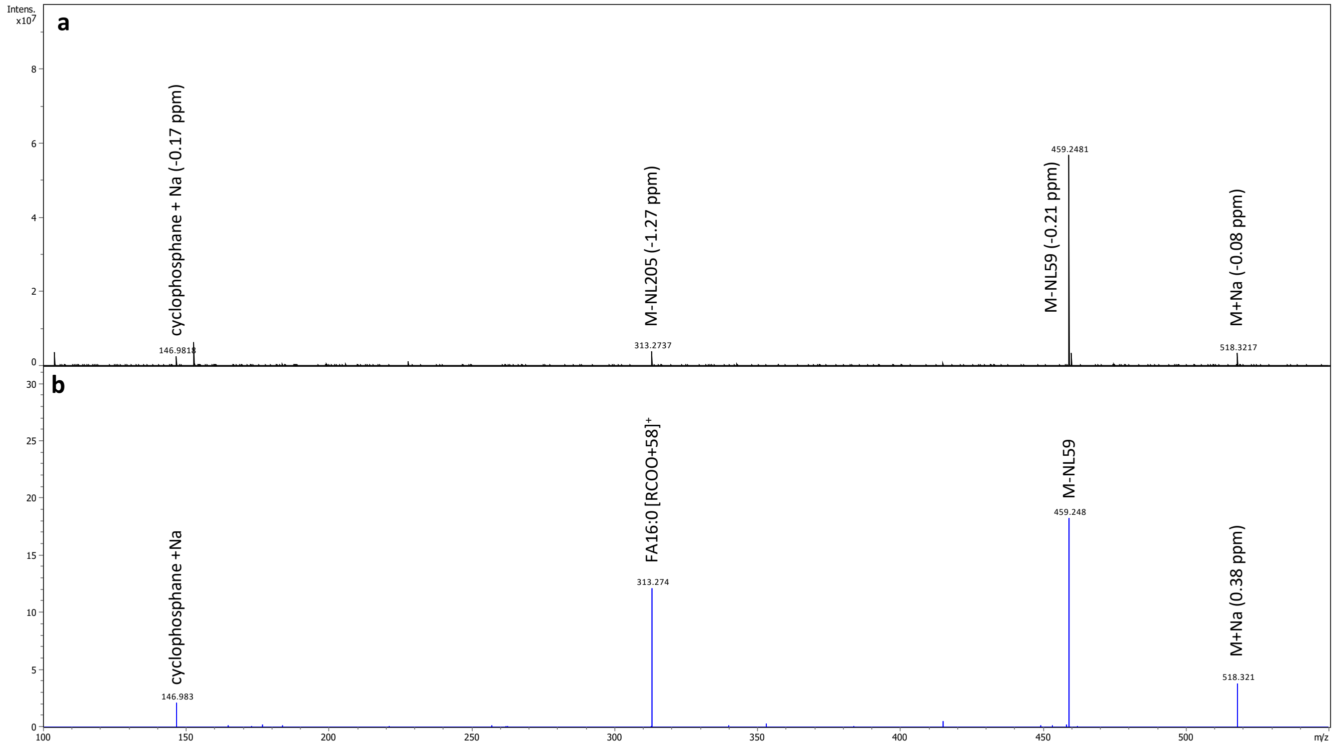

Figure S5. FTICR (a) and TimsTOF (b) MS/MS spectra of *m/z* 518.3217 [LPC 16:0+Na]$^+$

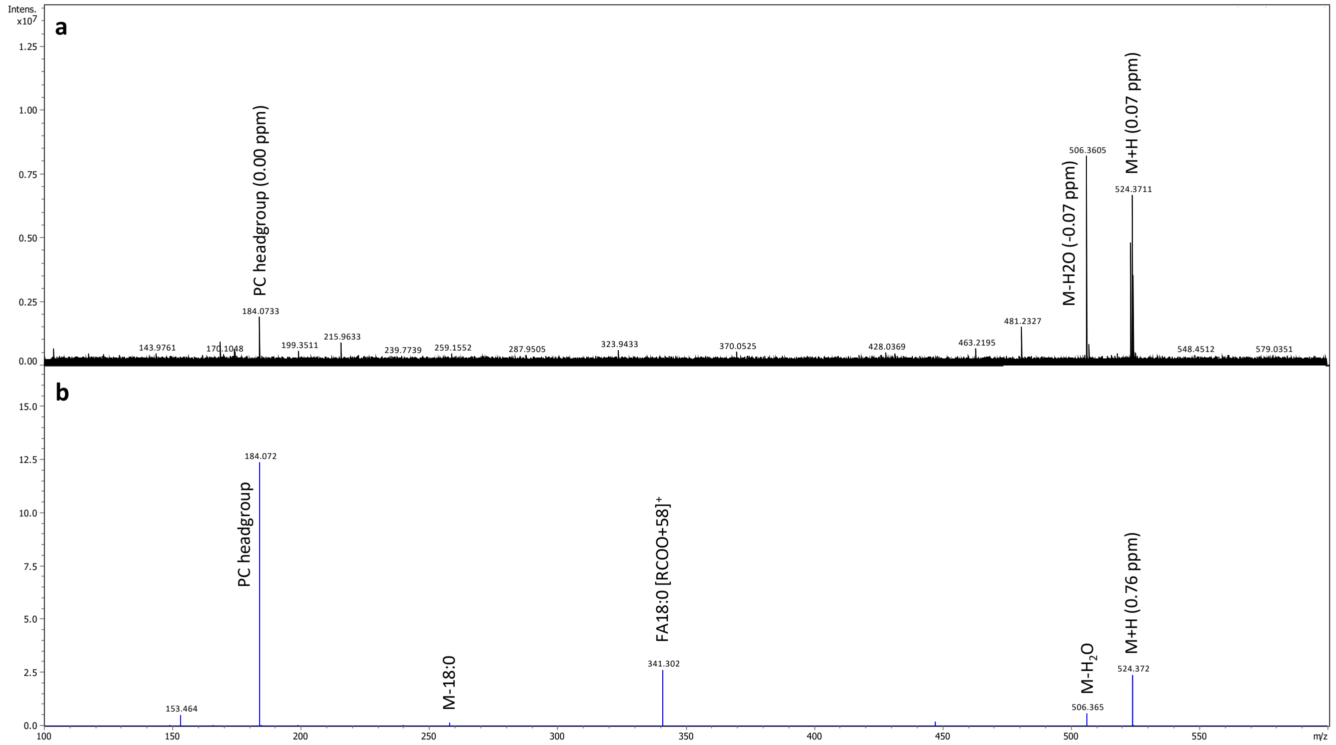

Figure S6. FTICR (a) and TimsTOF (b) MS/MS spectra of *m/z* 524.3711 [LPC 18:0]$^+$

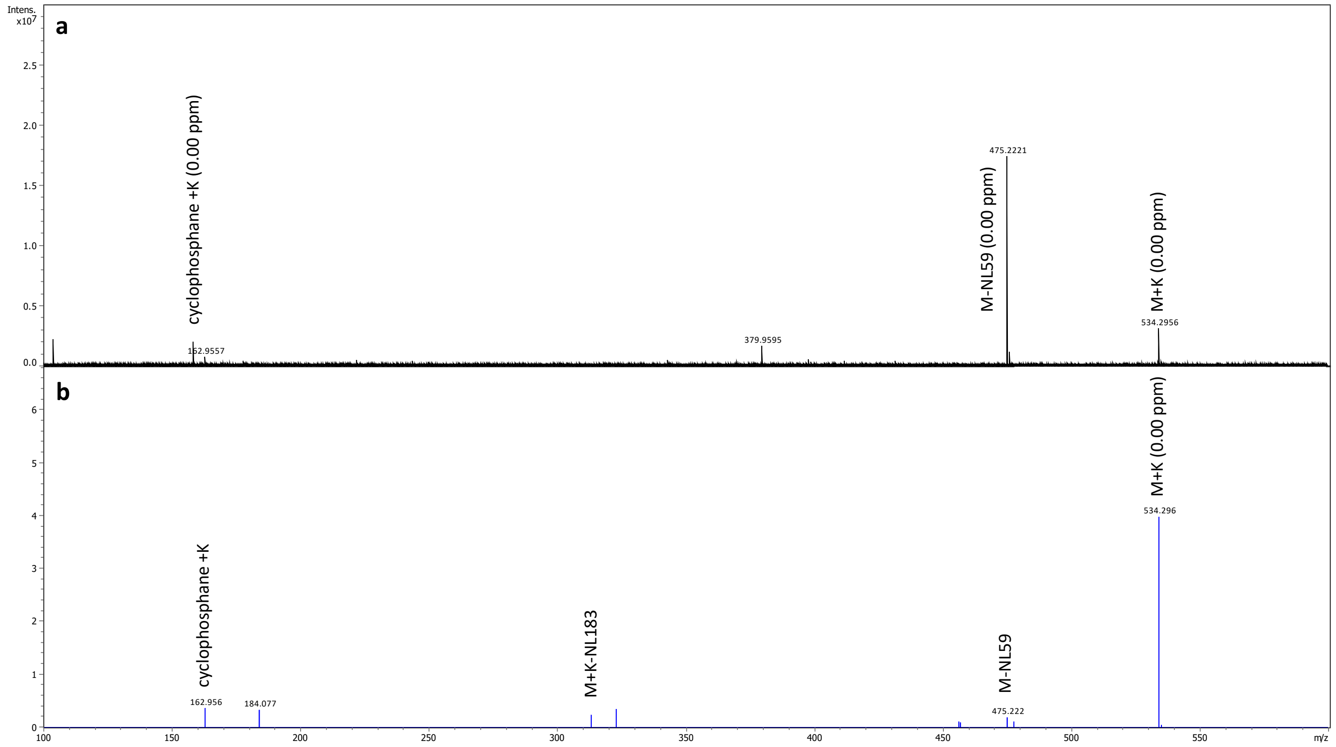

Figure S7. FTICR (a) and TimsTOF (b) MS/MS spectrum of *m/z* 534.2956 [LPC 16:0+K]$^+$

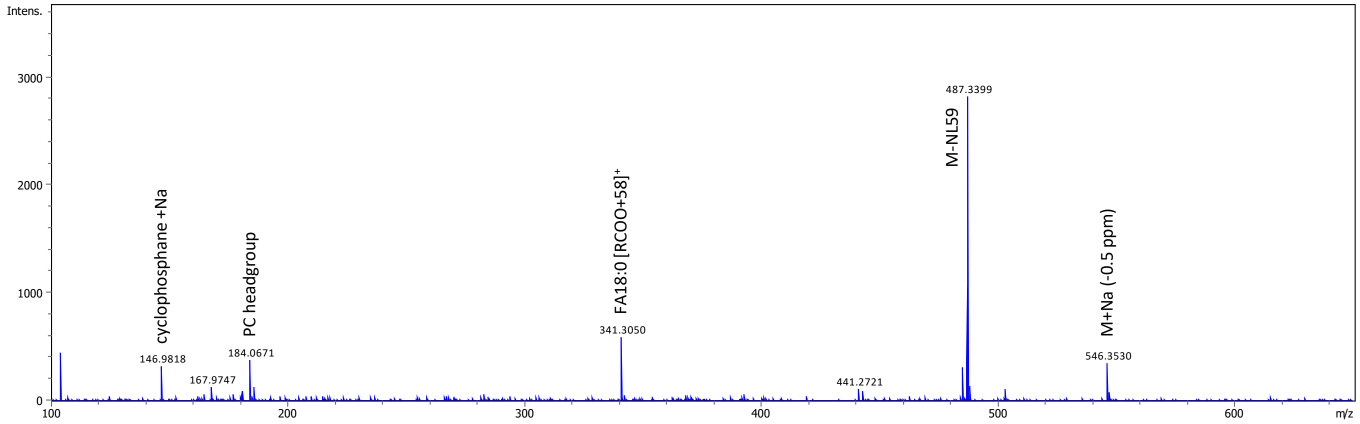

Figure S8. TimsTOF MS/MS spectrum of *m/z* 546.353 [LPC 18:0+Na]$^+$.

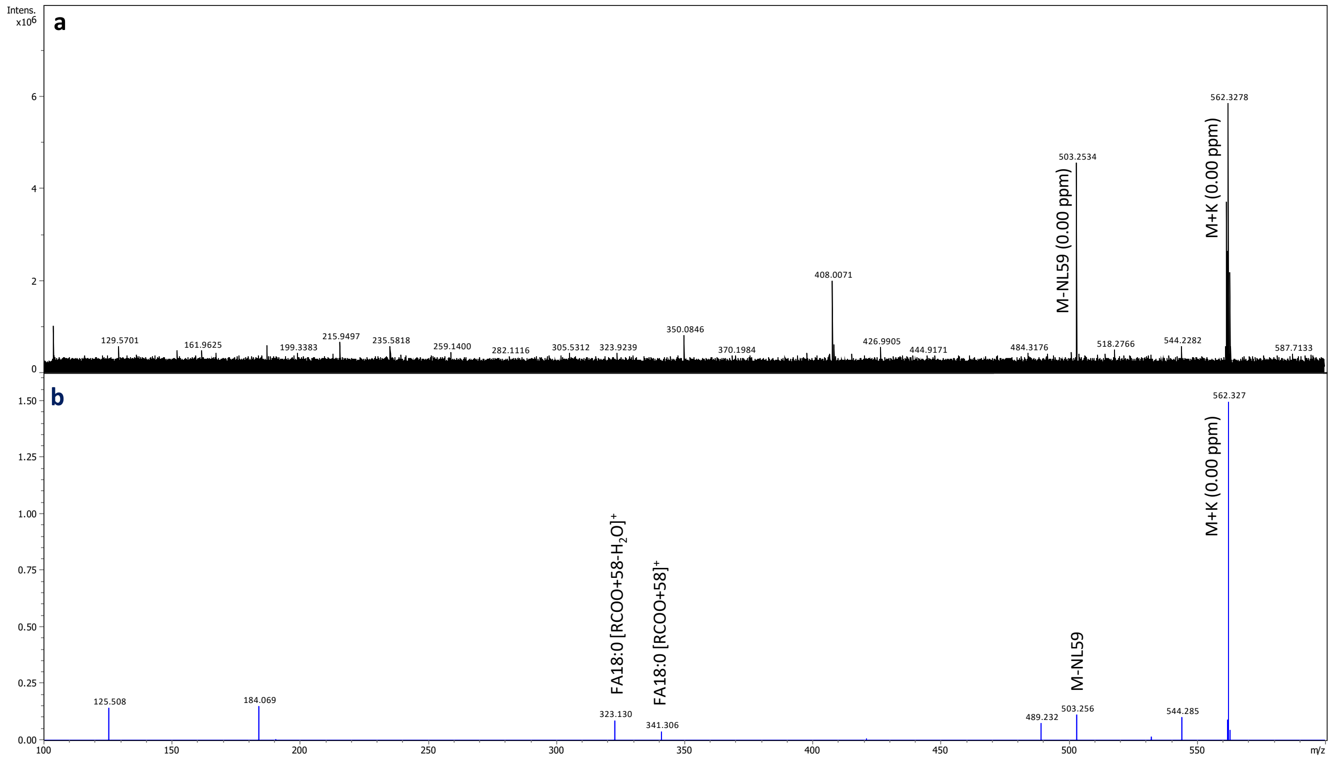

Figure S9. FTICR (a) and TimsTOF (b) MS/MS spectra of *m/z* 562.3278 [LPC 18:0+K]$^+$

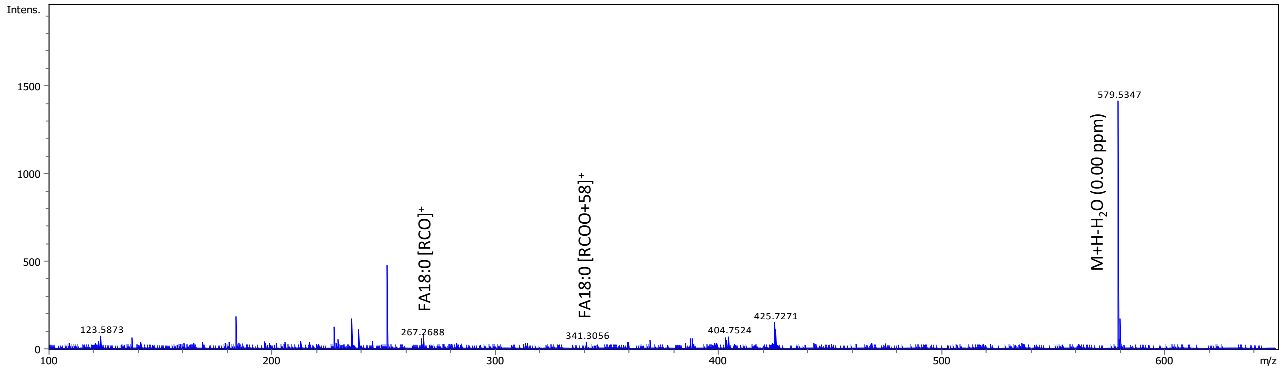

Figure S10. TimsTOF MS/MS spectrum of *m/z* 579.5345 [DG 34:0-H_2O]$^+$.

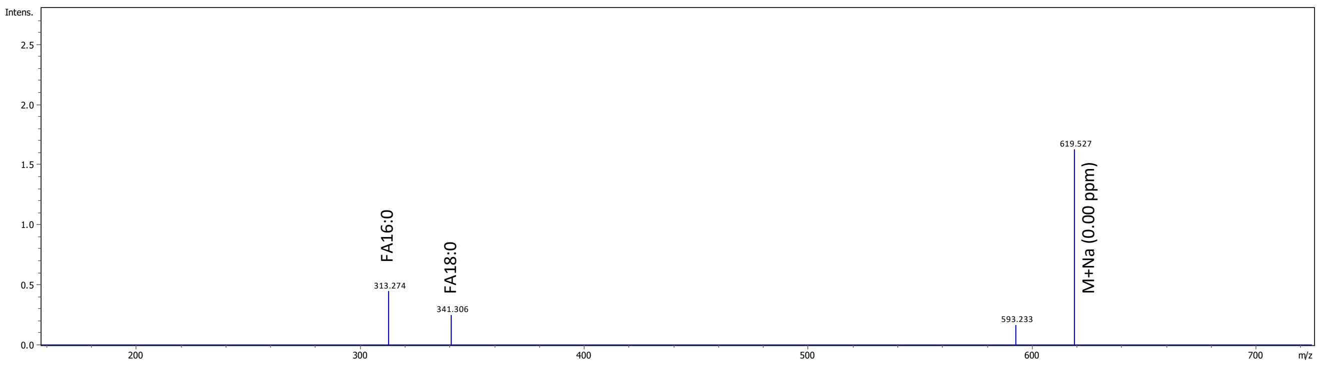

Figure S11. TimsTOF MS/MS spectrum of *m/z* 619.5267 [DG 34:0+Na]$^+$.

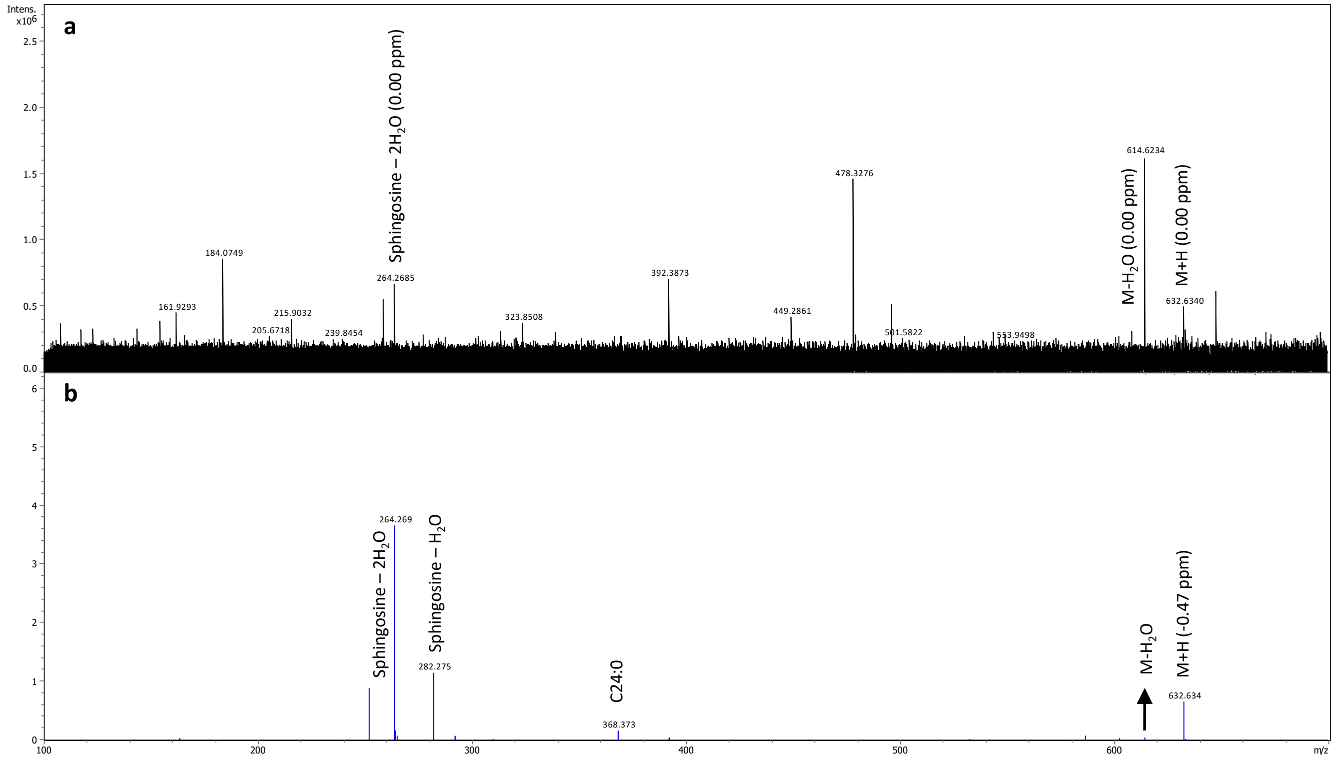

Figure S12. FTICR (a) and TimsTOF (b) MS/MS spectra of *m/z* 632.6340 [Cer d42:1-H_2O]$^+$

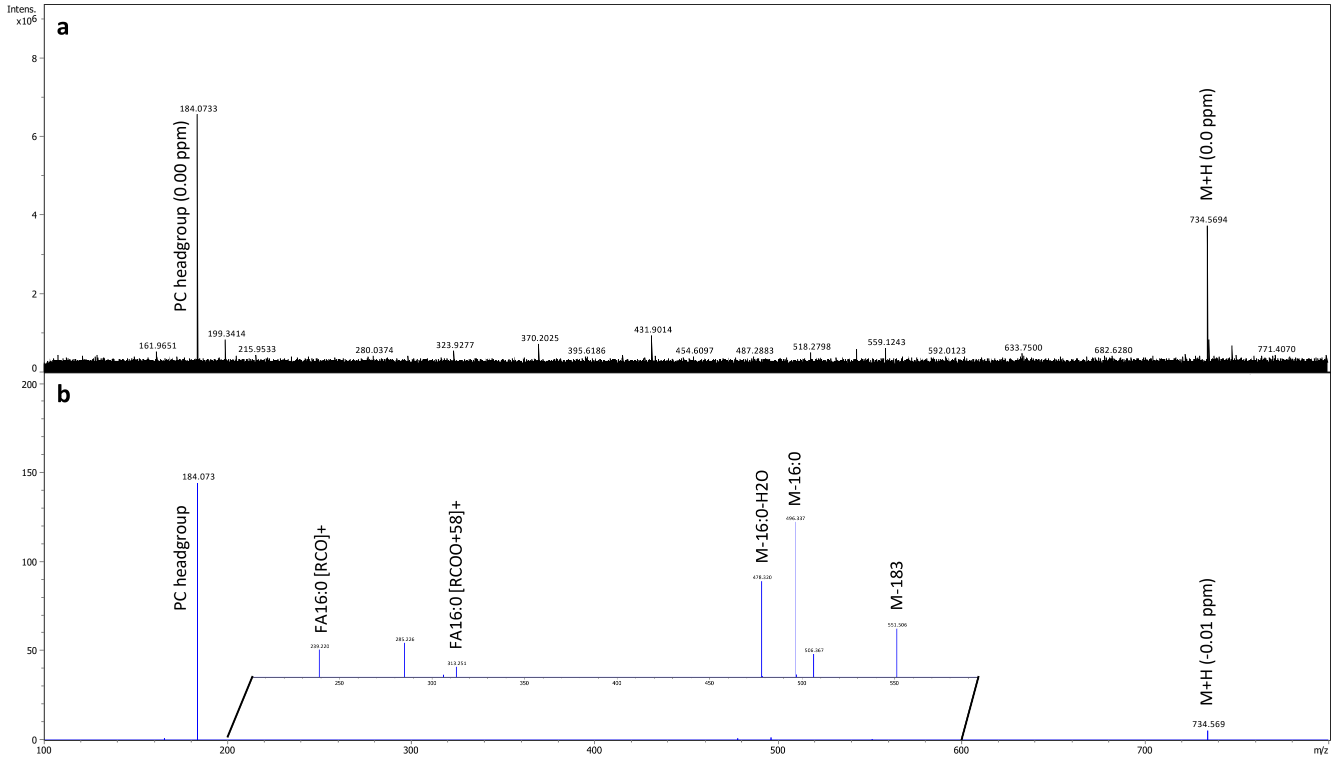

Figure S13. FTICR (a) and TimsTOF (b) MS/MS spectra of *m/z* 734.5694 [PC 32:0]$^+$

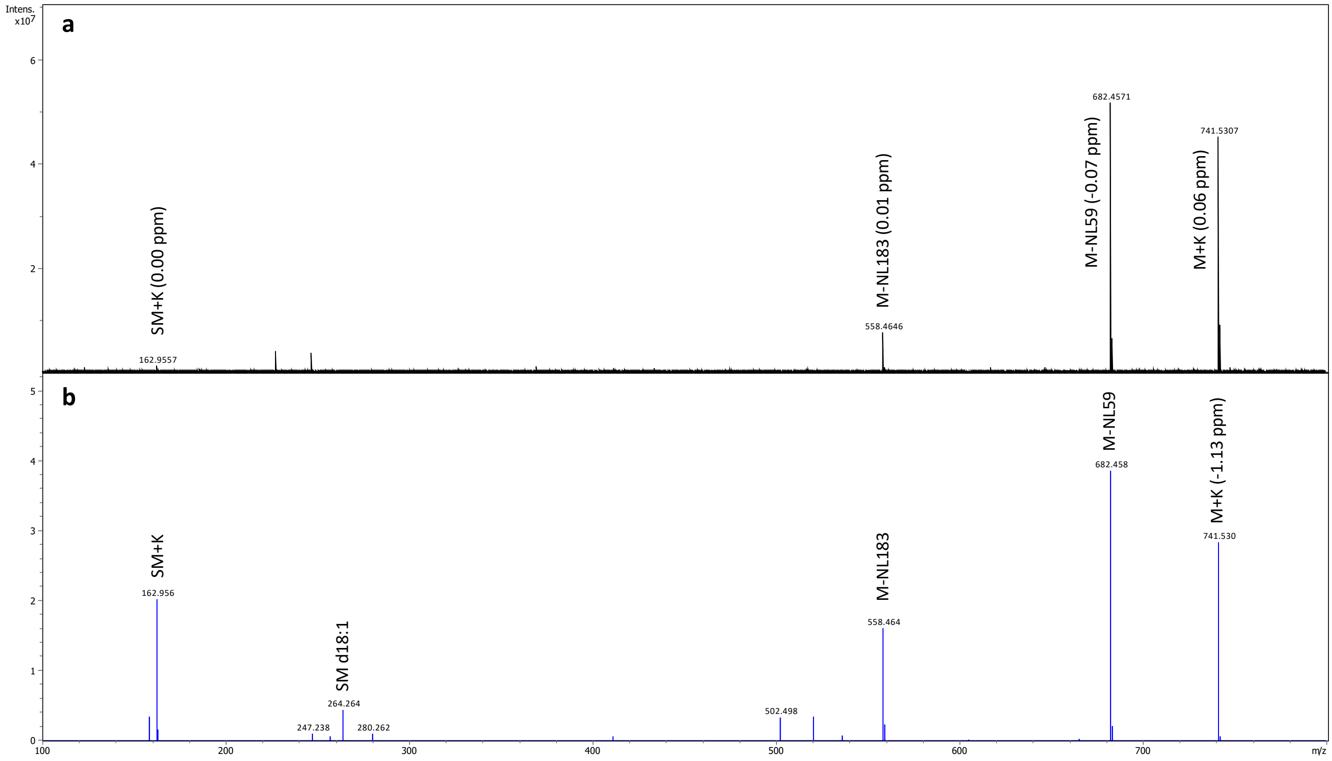

Figure S14. FTICR (a) and TimsTOF (b) MS/MS spectra of *m/z* 741.5307 [SM 34:1+K]$^+$

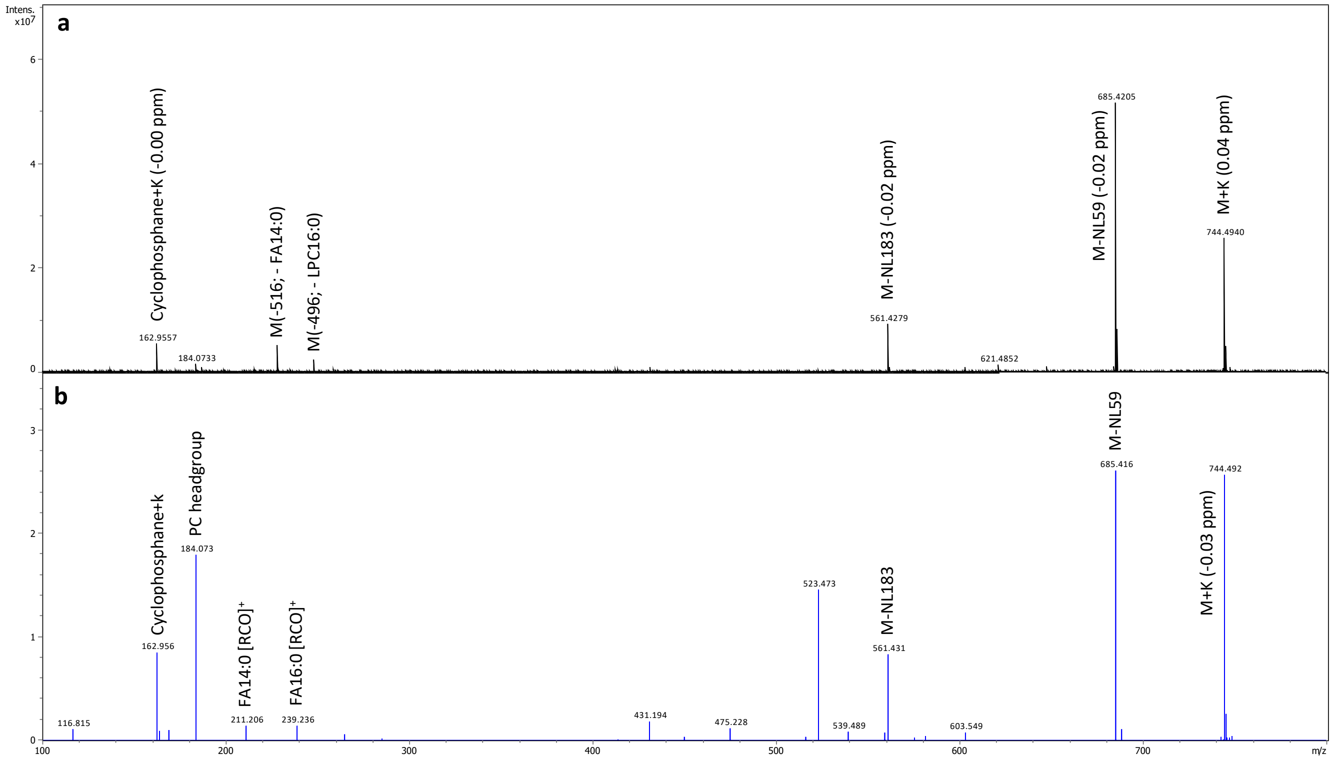

Figure S15. FTICR (a) and TimsTOF (b) MS/MS spectra of *m/z* 744.4940 [PC 30:0+K]$^+$

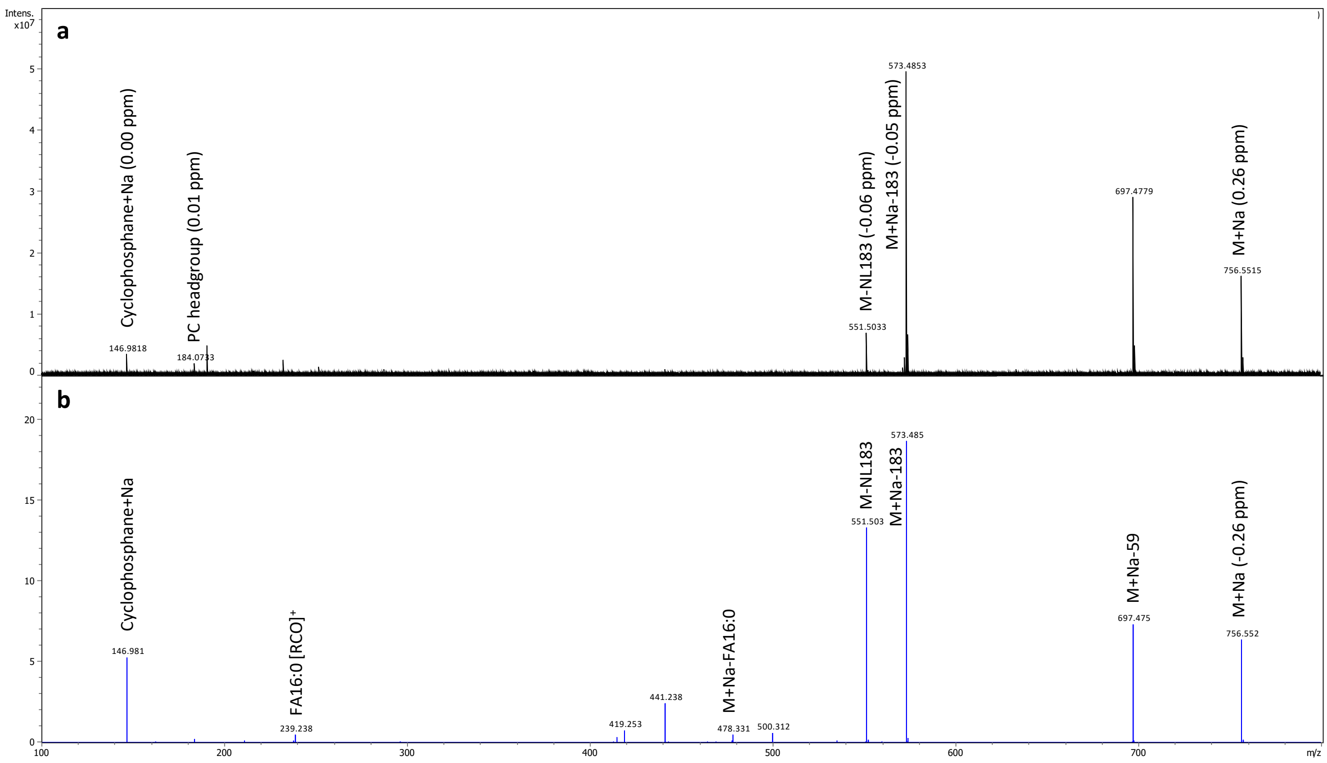

Figure S16. FTICR (a) and TimsTOF (b) MS/MS spectra of *m/z* 756.5515 [PC 32:0+Na]$^+$

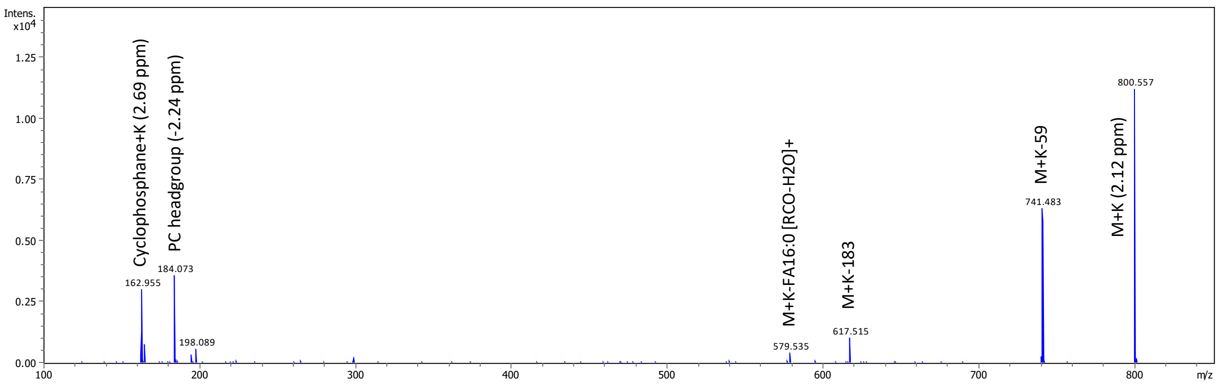

Figure S17. TimsTOF MS/MS spectrum of *m/z* 800.557 [PC 34:0+K]$^+$.

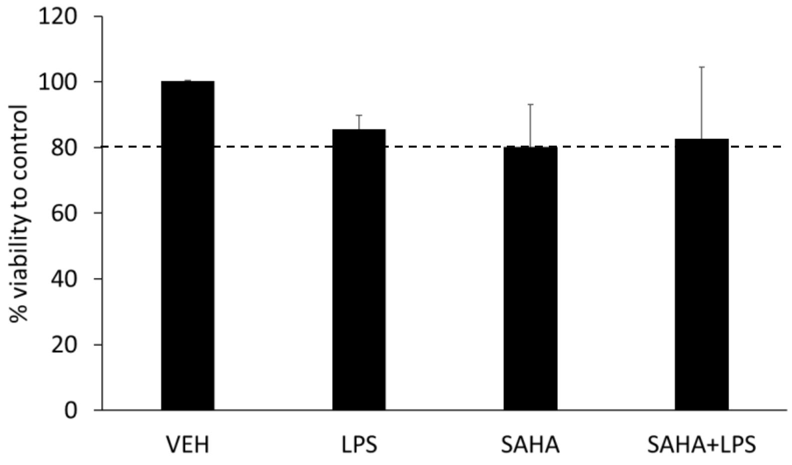

Figure S18. Percentage of viable cells after treatment with SAHA. SIM-A9 microglial cells were treated for 1h with SAHA or vehicle control (VEH) prior to LPS-stimulation for 18h (LPS 2.5 ng/ml). Cell viability was assessed by MTT assay. Data represent the mean of 3 biological replicates ± s.d. Data were statistically evaluated by one-way ANOVA with Tukey's post hoc test ($P = 0.3$).

Table ST1. Pearson's correlation for the vehicle-treated microglial cells MALDI-TOF MS fingerprinting in positive ion mode.

	VEH dataset1	VEH dataset2	VEH dataset3	VEH dataset4	VEH dataset5	VEH dataset6
**VEH dataset1**	-	0.91	0.91	0.99	0.98	0.96
**VEH dataset2**	0.91	-	0.98	0.90	0.87	0.84
**VEH dataset3**	0.91	0.98	-	0.88	0.87	0.84
**VEH dataset4**	0.99	0.90	0.88	-	0.97	0.96
**VEH dataset5**	0.98	0.87	0.87	0.97	-	0.97
**VEH dataset6**	0.96	0.84	0.84	0.96	0.97	

Table ST2. Pearson's correlation for the vehicle-treated microglial cells MALDI-TOF MS fingerprinting in negative ion mode.

	VEH dataset1	VEH dataset2	VEH dataset3	VEH dataset4
**VEH dataset1**	-	0.83	0.95	0.93
**VEH dataset2**	0.83	-	0.83	0.85
**VEH dataset3**	0.95	0.83	-	0.95
**VEH dataset4**	0.93	0.85	0.95	

Table ST3. . Significantly altered *m/z* features in LPS-treated microglial cells. Welch's T-Test and Benjamini & Hochberg adjusted *P* value threshold ≤ 0.01 for positive ion mode.

MALDI-TOF MS	LPS 2.5ng/ml		LPS 10ng/ml		LPS 100ng/ml	
m/z	log2FC	padj	log2FC	padj	log2FC	padj
391.32	0.6	0.051	0.7	0.003	0.7	0.044
412.36	0.8	0.016	0.9	0.019	0.9	0.011
429.28	0.6	0.042	0.9	0.003	0.8	0.001
435.31	0.8	0.017	0.8	0.003	0.8	0.012
449.29	1.3	0.007	1.4	0.001	1.4	0.002
468.31	0.9	0.003	0.9	0.009	0.8	0.004
482.36	0.8	0.008	0.9	0.005	0.8	0.010
494.33	0.8	0.003	0.7	0.008	0.4	0.054
496.35	1.2	0.001	1.2	<0.001	1.2	0.002
506.29	0.3	0.053	0.4	0.006	0.3	0.023
516.31	0.5	0.005	0.4	0.006	0.4	0.041
518.33	0.8	0.008	0.9	0.005	0.8	0.002
520.34	0.5	0.013	0.6	0.008	0.6	0.011
520.52	0.8	0.011	0.9	0.003	1.0	0.012
523.48	1.9	0.008	2.1	0.001	2.1	0.002
524.38	1.2	0.002	1.4	<0.0001	1.4	<0.0001
534.31	1.2	0.005	1.4	<0.001	1.3	0.002
537.54	1.6	0.008	1.7	0.003	1.7	0.013
538.54	1.3	0.008	1.4	0.003	1.3	0.009
546.37	0.9	0.010	1.0	0.001	1.1	<0.001
549.50	0.9	0.010	0.9	0.011	0.8	0.009
551.52	2.6	0.013	2.8	0.003	2.7	0.004
562.34	1.0	0.008	1.3	<0.001	1.3	0.001
563.48	1.3	0.002	1.4	0.002	1.6	0.011
565.57	1.3	0.008	1.4	0.004	1.3	0.009
577.53	1.1	0.008	1.0	0.011	1.0	0.016
579.55	2.2	0.011	2.3	0.005	2.3	0.003
591.52	1.8	0.007	2.0	0.001	2.1	0.011
619.55	1.7	0.004	1.8	0.001	1.8	0.005
620.55	1.5	0.007	1.5	0.001	1.6	0.006
632.66	0.8	0.007	0.8	0.008	0.8	0.003
633.64	0.9	0.005	0.8	0.007	0.8	0.003
672.64	0.3	0.003	0.3	0.021	0.3	0.012
698.46	-0.7	0.013	-0.9	0.002	-1.0	0.004
699.48	-0.6	0.024	-0.7	0.005	-0.7	0.005
708.56	0.4	0.029	0.4	0.018	0.5	0.011
722.57	0.7	0.002	0.7	0.001	0.9	0.005
723.57	0.6	0.001	0.6	<0.001	0.7	0.002
734.59	0.8	0.008	0.8	<0.001	0.8	0.003
736.59	0.7	0.002	0.8	<0.001	0.8	0.005
741.55	0.4	0.003	0.6	0.006	0.5	0.005
742.56	0.3	0.019	0.4	0.009	0.4	0.010
744.52	0.6	0.008	0.9	<0.001	0.8	0.003
756.57	0.6	0.009	0.7	0.005	0.7	0.005
763.61	0.6	0.012	0.6	0.009	0.5	0.005
772.55	0.9	<0.001	1.2	<0.001	1.1	<0.001
773.55	0.9	0.001	1.2	<0.001	1.1	0.002
800.57	0.7	0.003	0.8	0.003	0.7	0.008
829.74	1.7	0.002	2.0	<0.001	2.1	0.004
850.65	0.6	0.003	0.7	0.013	0.7	0.035
857.77	1.6	0.005	1.7	<0.001	1.8	0.002

Table ST4. Comparative CCS values of significantly altered *m/z* features in LPS-treated microglial cells.

Lipid assignment	LMSD_ID	TimsTOF fleX CCS Å^2	CCS Databases CCS Å^2	Literature CCS Å^2	mean ± s.d CCS values	Difference (%)
[LPC 14:0]$^+$	LMGP01050012	222.9	220.8[a] / 222.0[b] / 223.9[c] / 223.0[d]	223.1[e] / 233.0[f]	222.8 ± 1.0	0.04
[LPC O-16:0]$^+$	LMGP01060010	231.3	231.7[b] / 228.6[c] / 229.5[d]		229.9 ± 1.5	0.59
[LPC 16:0]$^+$	LMGP01050074	230.1	231.4[a] / 232.3[b] / 232.5[c] / 230.5[c] / 230.7[d]	231.0[e] / 229.9[e] / 225.7[f] / 231.1[f] / 231.2[h]	230.7 ± 1.9	-0.25
[LPC 16:0+Na]$^+$	LMGP01050018	233.1	234.3[a] / 233.9[b] / 234.2[c] / 235.7[d] / 223.9[d]		234.5 ± 0.8	-0.61
[LPC 18:0]$^+$	LMGP01050076	236.4	238.8[a] / 240.7[b] / 239.1[c] / 237.1[c] / 238.2[d]	238.4[e] / 237.1[e] / 232.9[f] / 238.5[f] / 239.0[h] / 240.2[h]	238.3 ± 2.1	-0.79
[LPC 18:0+Na]$^+$	LMGP01050076	239.2	241.5[a] / 242.3[b]/ 242.1[b] / 240.7[c] / 242.2[d]		241.8 ± 0.6	-1.06
[DG 34:0-H_2O]$^+$	LMGL02010003	263.1	268.0[b]		268.0 ± 0.0	-1.83
[DG 34:0+Na]$^+$	LMGL02010003	261.6	269.1[b] / 267.2[c]		268.2 ± 1.3	-2.44
[Cer d42:1-H_2O]$^+$	LMSP02010012	277.3	277.2[a] / 283.0[b] / 282.6[c] / 275.0[d]	275.0[g]	278.6 ± 3.9	-0.46
[PC 32:0]$^+$	LMGP01010564	282.8	284.6[a] / 286.6[b] / 290.2[b] / 284.9[c] / 281.9[d]	278.4[g] / 288.6[h]	284.4 ± 4.0	-0.58
[PC 32:0+Na]$^+$	LMGP01010564	287.9	281.7[a] / 290.3[b] / 286.4[c] / 279.7[d]	280.5[g]	283.7 ± 4.4	1.47

References

[a]Picache, J. A. et al. Collision cross section compendium to annotate and predict multi-omic compound identities. Chem. Sci. 10, 983–993 (2019).

[b]Zhou, Z. et al. Ion mobility collision cross-section atlas for known and unknown metabolite annotation in untargeted metabolomics. Nat. Commun. 11, 4334 (2020).

[c]Zhou, Z., Tu, J., Xiong, X., Shen, X. & Zhu, Z.-J. LipidCCS: Prediction of Collision Cross-Section Values for Lipids with High Precision To Support Ion Mobility–Mass Spectrometry-Based Lipidomics. Anal. Chem. 89, 9559–9566 (2017).

[d]Ross, D. H., Cho, J. H. & Xu, L. Breaking Down Structural Diversity for Comprehensive Prediction of Ion-Neutral Collision Cross Sections. Anal. Chem. 92, 4548–4557 (2020).

[e]Vasilopoulou, C. G. et al. Trapped ion mobility spectrometry and PASEF enable in-depth lipidomics from minimal sample amounts. Nat. Commun. 11, 331 (2020).

[f]Tsugawa, H. et al. A lipidome atlas in MS-DIAL 4. Nat. Biotechnol. 38, 1159–1163 (2020).

[g]Leaptrot, K. L., May, J. C., Dodds, J. N. & McLean, J. A. Ion mobility conformational lipid atlas for high confidence lipidomics. Nat. Commun. 10, 985 (2019)

[h]Helmer, P. O. et al. Complementing Matrix-Assisted Laser Desorption Ionization-Mass Spectrometry Imaging with Chromatography Data for Improved Assignment of Isobaric and Isomeric Phospholipids Utilizing Trapped Ion Mobility-Mass Spectrometry. Anal. Chem. 93, 2135–2143 (2021).
